# Interval estimation and optimal design for the within-subject coefficient of variation for continuous and binary variables

**DOI:** 10.1186/1471-2288-6-24

**Published:** 2006-05-10

**Authors:** Mohamed M Shoukri, Nasser Elkum, Stephen D Walter

**Affiliations:** 1 Department of Biostatistics, Epidemiology and Scientific Computing King Faisal Specialist Hospital and Research Centre, P.O. Box 3354, Riyadh 11211, Saudi Arabia; 2 Department of Epidemiology and Biostatistics, University of Western Ontario, London, Ontario, Canada; 3 Department of Epidemiology and Biostatistics, McMaster University Hamilton, Ontario, Canada

## Abstract

**Background:**

In this paper we propose the use of the within-subject coefficient of variation as an index of a measurement's reliability. For continuous variables and based on its maximum likelihood estimation we derive a variance-stabilizing transformation and discuss confidence interval construction within the framework of a one-way random effects model. We investigate sample size requirements for the within-subject coefficient of variation for continuous and binary variables.

**Methods:**

We investigate the validity of the approximate normal confidence interval by Monte Carlo simulations. In designing a reliability study, a crucial issue is the balance between the number of subjects to be recruited and the number of repeated measurements per subject. We discuss efficiency of estimation and cost considerations for the optimal allocation of the sample resources. The approach is illustrated by an example on Magnetic Resonance Imaging (MRI). We also discuss the issue of sample size estimation for dichotomous responses with two examples.

**Results:**

For the continuous variable we found that the variance stabilizing transformation improves the asymptotic coverage probabilities on the within-subject coefficient of variation for the continuous variable. The maximum like estimation and sample size estimation based on pre-specified width of confidence interval are novel contribution to the literature for the binary variable.

**Conclusion:**

Using the sample size formulas, we hope to help clinical epidemiologists and practicing statisticians to efficiently design reliability studies using the within-subject coefficient of variation, whether the variable of interest is continuous or binary.

## Background

Measurement errors can seriously affect statistical analysis and interpretation; it therefore becomes important to assess the magnitude of such errors by calculating a reliability coefficient and assessing its precision. For instance medical diagnosis, clinicians have now become cognizant to the paramount importance of obtaining accurate measurements to ensure safe and efficient delivery of care to their patients. Experiments designed to measure validity and precision of instruments used in biomedical and epidemiological research are ubiquitous. For example, Ashton [1] demonstrated the importance of evaluating the reliability of manual and automated methods for quantifying total white matter lesions burden in multiple sclerosis patients. They compared the coefficient of variations of three methods. In oncology, Schwartz et al. [2] used the coefficient of variation to evaluate the repeatability in bi-dimensional computed tomography measurements of three techniques: hand-held calipers on film, electronic calipers on a workstation, and an auto-contour technique on a workstation. Differences between the coefficients of variation were statistically significantly different for the auto-contour technique, compared to the other techniques. The coefficient of variation is often used to compare variables measured on different scales. For example, in social sciences, when the intent is to compare the variability in school performance with the variability of household income, a comparison of standard deviations makes no sense because income and school performance are measured on different scales. The correct comparison may be based on the coefficient of variation because it adjusts for scale. Other applications of the coefficient of variation are given in Tian [3].

Scientists have developed several indices to assess the reliability and reproducibility of quantitative measurements. The intra-class correlation (ICC), the proportion of the between-subject variance to the total variance, has been widely used as an index of measurement reliability. For a comprehensive review on the ICC and its applications, we refer the reader to Fleiss [4], Dunn [5] and Shoukri [6]. One of the criticisms of the ICC is that its value depends on the population from which the study subjects have been obtained, and this may lead to difficulties in comparing results from different studies. Accordingly, Quan and Shih [7] (QS) considered an alternative measure, the Within-Subject Coefficient of Variation (WSCV) as an alternative to the ICC for assessing measurements reproducibility or test-re-test reliability. Because of the requirement that repeated observations are made on each subject, they used the one-way random effects model (REM) as a mechanism to describe the data. Although the use of the WSCV as a measure of reproducibility is long standing, the issue of sample size determination has not been adequately investigated. Sample size estimation is one of the most important issues in the design of any study that uses inferential statistics.

When the ICC is used as the index of reliability, Donner and Eliasziw [8] provided contours of exact power for selected numbers of subjects (*k*) and numbers of replicates (*n*). These power results were then used to identify optimal designs that minimize the study costs. Assuming a constant number of replicates per subject, Walter et al. [9] considered an approximation to determine the required number of subjects to achieve fixed levels of power. Bonett [10] calculated the sample size required to achieve a prescribed expected width for the confidence interval on the ICC. Shoukri et al. [11] derived the values of *k *and *n *that allocate the sample resources optimally and minimize the variance of the estimated ICC under cost constraints. The cost structure that was considered was general and followed the general guidelines identified by Flynn et al. [12].

In this paper, we derive the optimal allocation for the number of subjects and the number of repeated measurements needed to minimize the variance of the maximum likelihood estimator (MLE) of the WSCV. In Section 2 we present the random effects model, the definition of the WSCV, and the asymptotic distribution of its MLE for continuous data. In Section 3, we use the calculus of optimization to find the optimal combinations (*n*, *k*) that minimize the variance of the MLE of WSCV for normally distributed variables. The use of the WSCV for dichotomous data has never been investigated before, and a novel contribution in this paper is the estimation of WSCV for binary outcome measurements, and sample size requirements, with emphasis on the case of two ratings per subject (i.e. *n *= 2). We devote Section 4 to the binary data, and general discussion is presented in Section 5.

## Methods

### Estimating the WSCV for continuous variables

#### Assumptions

Consider a random sample of *k *subjects with *n *repeated measurements of a continuous variable *Y*, and denote by *Y*_*ij *_the *j*^*th *^reading made on the *i*^*th *^subject under identical experimental conditions (*i *= 1,2,...*k*; *j *= 1,2,...*n*). In a test-retest scenario, and under the assumption of no reader's effect (*i.e*. the readings within a specific subject are exchangeable), *Y*_*ij *_denotes the reading of the *j*^*th *^trial made on the *i*^*th *^subject. A useful model for analyzing such data is given by:

*Y*_*ij *_= *μ *+ *s*_*i *_+ *e*_*ij *_    *i *= 1,2,...*k*; *j *= 1,2,...*n *    (1)

where *μ *is the mean of *Y*_*ij*_, the random subject effects *s*_*i *_are normally distributed with mean 0 and variance σs2
 MathType@MTEF@5@5@+=feaafiart1ev1aaatCvAUfKttLearuWrP9MDH5MBPbIqV92AaeXatLxBI9gBaebbnrfifHhDYfgasaacH8akY=wiFfYdH8Gipec8Eeeu0xXdbba9frFj0=OqFfea0dXdd9vqai=hGuQ8kuc9pgc9s8qqaq=dirpe0xb9q8qiLsFr0=vr0=vr0dc8meaabaqaciaacaGaaeqabaqabeGadaaakeaaiiGacqWFdpWCdaqhaaWcbaGaem4CamhabaGaeGOmaidaaaaa@3104@ , or *N*(0, σs2
 MathType@MTEF@5@5@+=feaafiart1ev1aaatCvAUfKttLearuWrP9MDH5MBPbIqV92AaeXatLxBI9gBaebbnrfifHhDYfgasaacH8akY=wiFfYdH8Gipec8Eeeu0xXdbba9frFj0=OqFfea0dXdd9vqai=hGuQ8kuc9pgc9s8qqaq=dirpe0xb9q8qiLsFr0=vr0=vr0dc8meaabaqaciaacaGaaeqabaqabeGadaaakeaaiiGacqWFdpWCdaqhaaWcbaGaem4CamhabaGaeGOmaidaaaaa@3104@), the measurement errors *e*_*ij *_are *N*(0, σe2
 MathType@MTEF@5@5@+=feaafiart1ev1aaatCvAUfKttLearuWrP9MDH5MBPbIqV92AaeXatLxBI9gBaebbnrfifHhDYfgasaacH8akY=wiFfYdH8Gipec8Eeeu0xXdbba9frFj0=OqFfea0dXdd9vqai=hGuQ8kuc9pgc9s8qqaq=dirpe0xb9q8qiLsFr0=vr0=vr0dc8meaabaqaciaacaGaaeqabaqabeGadaaakeaaiiGacqWFdpWCdaqhaaWcbaGaemyzaugabaGaeGOmaidaaaaa@30E8@), and the *s*_*i *_and *e*_*ij*_terms are independent. We assume that the subjects are randomly drawn from some population of interest.

Quan and Shih [7] defined the WSCV parameter in the above model as:

*θ *= *σ*_*e*_/*μ *    (2)

With model (1), it is assumed that the within subject variance is the same for all subjects.

### Maximum likelihood estimator

Under the above set-up, the log-likelihood function has the form

l=−nk2log⁡2π−12k(n−1)log⁡σe2−12k[log⁡(σe2/n+σs2)]−∑i∑j(yij−μ)22σe2+n2σs2∑i(y¯i−μ)22σe2(σe2+nσs2).
 MathType@MTEF@5@5@+=feaafiart1ev1aaatCvAUfKttLearuWrP9MDH5MBPbIqV92AaeXatLxBI9gBaebbnrfifHhDYfgasaacH8akY=wiFfYdH8Gipec8Eeeu0xXdbba9frFj0=OqFfea0dXdd9vqai=hGuQ8kuc9pgc9s8qqaq=dirpe0xb9q8qiLsFr0=vr0=vr0dc8meaabaqaciaacaGaaeqabaqabeGadaaakeaacqWGSbaBcqGH9aqpcqGHsisldaWcaaqaaiabd6gaUjabdUgaRbqaaiabikdaYaaacyGGSbaBcqGGVbWBcqGGNbWzcqaIYaGmiiGacqWFapaCcqGHsisldaWcaaqaaiabigdaXaqaaiabikdaYaaacqWGRbWAcqGGOaakcqWGUbGBcqGHsislcqaIXaqmcqGGPaqkcyGGSbaBcqGGVbWBcqGGNbWzcqWFdpWCdaqhaaWcbaGaemyzaugabaGaeGOmaidaaOGaeyOeI0YaaSaaaeaacqaIXaqmaeaacqaIYaGmaaGaem4AaS2aamWaaeaacyGGSbaBcqGGVbWBcqGGNbWzdaqadaqaaiab=n8aZnaaDaaaleaacqWGLbqzaeaacqaIYaGmaaGccqGGVaWlcqWGUbGBcqGHRaWkcqWFdpWCdaqhaaWcbaGaem4CamhabaGaeGOmaidaaaGccaGLOaGaayzkaaaacaGLBbGaayzxaaGaeyOeI0YaaSaaaeaadaaeqbqaamaaqafabaWaaeWaaeaacqWG5bqEdaWgaaWcbaGaemyAaKMaemOAaOgabeaakiabgkHiTiab=X7aTbGaayjkaiaawMcaaaWcbaGaemOAaOgabeqdcqGHris5aOWaaWbaaSqabeaacqaIYaGmaaaabaGaemyAaKgabeqdcqGHris5aaGcbaGaeGOmaiJae83Wdm3aa0baaSqaaiabdwgaLbqaaiabikdaYaaaaaGccqGHRaWkdaWcaaqaaiabd6gaUnaaCaaaleqabaGaeGOmaidaaOGae83Wdm3aa0baaSqaaiabdohaZbqaaiabikdaYaaakmaaqafabaWaaeWaaeaacuWG5bqEgaqeamaaBaaaleaacqWGPbqAaeqaaOGaeyOeI0Iae8hVd0gacaGLOaGaayzkaaWaaWbaaSqabeaacqaIYaGmaaaabaGaemyAaKgabeqdcqGHris5aaGcbaGaeGOmaiJae83Wdm3aa0baaSqaaiabdwgaLbqaaiabikdaYaaakmaabmaabaGae83Wdm3aa0baaSqaaiabdwgaLbqaaiabikdaYaaakiabgUcaRiabd6gaUjab=n8aZnaaDaaaleaacqWGZbWCaeaacqaIYaGmaaaakiaawIcacaGLPaaaaaGaeiOla4caaa@A0C4@

Define *σ*^2 ^= σs2
 MathType@MTEF@5@5@+=feaafiart1ev1aaatCvAUfKttLearuWrP9MDH5MBPbIqV92AaeXatLxBI9gBaebbnrfifHhDYfgasaacH8akY=wiFfYdH8Gipec8Eeeu0xXdbba9frFj0=OqFfea0dXdd9vqai=hGuQ8kuc9pgc9s8qqaq=dirpe0xb9q8qiLsFr0=vr0=vr0dc8meaabaqaciaacaGaaeqabaqabeGadaaakeaaiiGacqWFdpWCdaqhaaWcbaGaem4CamhabaGaeGOmaidaaaaa@3104@ + σe2
 MathType@MTEF@5@5@+=feaafiart1ev1aaatCvAUfKttLearuWrP9MDH5MBPbIqV92AaeXatLxBI9gBaebbnrfifHhDYfgasaacH8akY=wiFfYdH8Gipec8Eeeu0xXdbba9frFj0=OqFfea0dXdd9vqai=hGuQ8kuc9pgc9s8qqaq=dirpe0xb9q8qiLsFr0=vr0=vr0dc8meaabaqaciaacaGaaeqabaqabeGadaaakeaaiiGacqWFdpWCdaqhaaWcbaGaemyzaugabaGaeGOmaidaaaaa@30E8@ and *ρ *= σs2
 MathType@MTEF@5@5@+=feaafiart1ev1aaatCvAUfKttLearuWrP9MDH5MBPbIqV92AaeXatLxBI9gBaebbnrfifHhDYfgasaacH8akY=wiFfYdH8Gipec8Eeeu0xXdbba9frFj0=OqFfea0dXdd9vqai=hGuQ8kuc9pgc9s8qqaq=dirpe0xb9q8qiLsFr0=vr0=vr0dc8meaabaqaciaacaGaaeqabaqabeGadaaakeaaiiGacqWFdpWCdaqhaaWcbaGaem4CamhabaGaeGOmaidaaaaa@3104@/(σs2
 MathType@MTEF@5@5@+=feaafiart1ev1aaatCvAUfKttLearuWrP9MDH5MBPbIqV92AaeXatLxBI9gBaebbnrfifHhDYfgasaacH8akY=wiFfYdH8Gipec8Eeeu0xXdbba9frFj0=OqFfea0dXdd9vqai=hGuQ8kuc9pgc9s8qqaq=dirpe0xb9q8qiLsFr0=vr0=vr0dc8meaabaqaciaacaGaaeqabaqabeGadaaakeaaiiGacqWFdpWCdaqhaaWcbaGaem4CamhabaGaeGOmaidaaaaa@3104@ + σe2
 MathType@MTEF@5@5@+=feaafiart1ev1aaatCvAUfKttLearuWrP9MDH5MBPbIqV92AaeXatLxBI9gBaebbnrfifHhDYfgasaacH8akY=wiFfYdH8Gipec8Eeeu0xXdbba9frFj0=OqFfea0dXdd9vqai=hGuQ8kuc9pgc9s8qqaq=dirpe0xb9q8qiLsFr0=vr0=vr0dc8meaabaqaciaacaGaaeqabaqabeGadaaakeaaiiGacqWFdpWCdaqhaaWcbaGaemyzaugabaGaeGOmaidaaaaa@30E8@), the intra-class correlation coefficient, for which σs2
 MathType@MTEF@5@5@+=feaafiart1ev1aaatCvAUfKttLearuWrP9MDH5MBPbIqV92AaeXatLxBI9gBaebbnrfifHhDYfgasaacH8akY=wiFfYdH8Gipec8Eeeu0xXdbba9frFj0=OqFfea0dXdd9vqai=hGuQ8kuc9pgc9s8qqaq=dirpe0xb9q8qiLsFr0=vr0=vr0dc8meaabaqaciaacaGaaeqabaqabeGadaaakeaaiiGacqWFdpWCdaqhaaWcbaGaem4CamhabaGaeGOmaidaaaaa@3104@ = *ρσ*^2 ^and σe2
 MathType@MTEF@5@5@+=feaafiart1ev1aaatCvAUfKttLearuWrP9MDH5MBPbIqV92AaeXatLxBI9gBaebbnrfifHhDYfgasaacH8akY=wiFfYdH8Gipec8Eeeu0xXdbba9frFj0=OqFfea0dXdd9vqai=hGuQ8kuc9pgc9s8qqaq=dirpe0xb9q8qiLsFr0=vr0=vr0dc8meaabaqaciaacaGaaeqabaqabeGadaaakeaaiiGacqWFdpWCdaqhaaWcbaGaemyzaugabaGaeGOmaidaaaaa@30E8@ = *σ*^2^(1-*ρ*). Because the design is balanced the maximum likelihood estimators (MLE) for *μ*, *σ*^2^, and *ρ *are given in closed forms by: μ^=k−1∑i=1ky¯i, θ^=ΜSW/μ^
 MathType@MTEF@5@5@+=feaafiart1ev1aaatCvAUfKttLearuWrP9MDH5MBPbIqV92AaeXatLxBI9gBaebbnrfifHhDYfgasaacH8akY=wiFfYdH8Gipec8Eeeu0xXdbba9frFj0=OqFfea0dXdd9vqai=hGuQ8kuc9pgc9s8qqaq=dirpe0xb9q8qiLsFr0=vr0=vr0dc8meaabaqaciaacaGaaeqabaqabeGadaaakeaaiiGacuWF8oqBgaqcaiabg2da9iabdUgaRnaaCaaaleqabaGaeyOeI0IaeGymaedaaOWaaabCaeaacuWG5bqEgaqeamaaBaaaleaacqWGPbqAaeqaaOGaeiilaWcaleaacqWGPbqAcqGH9aqpcqaIXaqmaeaacqWGRbWAa0GaeyyeIuoakiaaykW7cuWF4oqCgaqcaiab=1da9maakaaabaGae8hNd0Kaem4uamLaem4vaCfaleqaaOGaei4la8Iaf8hVd0MbaKaaaaa@48E0@, σs2=(k−1)MSB−(k)MSWnk
 MathType@MTEF@5@5@+=feaafiart1ev1aaatCvAUfKttLearuWrP9MDH5MBPbIqV92AaeXatLxBI9gBaebbnrfifHhDYfgasaacH8akY=wiFfYdH8Gipec8Eeeu0xXdbba9frFj0=OqFfea0dXdd9vqai=hGuQ8kuc9pgc9s8qqaq=dirpe0xb9q8qiLsFr0=vr0=vr0dc8meaabaqaciaacaGaaeqabaqabeGadaaakeaaiiGacqWFdpWCdaqhaaWcbaGaem4CamhabaGaeGOmaidaaOGaeyypa0ZaaSaaaeaacqGGOaakcqWGRbWAcqGHsislcqaIXaqmcqGGPaqkcqWGnbqtcqWGtbWucqWGcbGqcqGHsislcqGGOaakcqWGRbWAcqGGPaqkcqWGnbqtcqWGtbWucqWGxbWvaeaacqWGUbGBcqWGRbWAaaaaaa@44BC@, and the estimated ICC is p⌢=(k−1)MSB−(k)MSW(k−1)MSB+k(n−1)MSW
 MathType@MTEF@5@5@+=feaafiart1ev1aaatCvAUfKttLearuWrP9MDH5MBPbIqV92AaeXatLxBI9gBaebbnrfifHhDYfgasaacH8akY=wiFfYdH8Gipec8Eeeu0xXdbba9frFj0=OqFfea0dXdd9vqai=hGuQ8kuc9pgc9s8qqaq=dirpe0xb9q8qiLsFr0=vr0=vr0dc8meaabaqaciaacaGaaeqabaqabeGadaaakeaacuWGWbaCgaWeaiabg2da9maalaaabaGaeiikaGIaem4AaSMaeyOeI0IaeGymaeJaeiykaKIaemyta0Kaem4uamLaemOqaiKaeyOeI0IaeiikaGIaem4AaSMaeiykaKIaemyta0Kaem4uamLaem4vaCfabaGaeiikaGIaem4AaSMaeyOeI0IaeGymaeJaeiykaKIaemyta0Kaem4uamLaemOqaiKaey4kaSIaem4AaSMaeiikaGIaemOBa4MaeyOeI0IaeGymaeJaeiykaKIaemyta0Kaem4uamLaem4vaCfaaaaa@5224@, where MSW=1k(n−1)∑ik∑jn(yij−y¯i)2
 MathType@MTEF@5@5@+=feaafiart1ev1aaatCvAUfKttLearuWrP9MDH5MBPbIqV92AaeXatLxBI9gBaebbnrfifHhDYfgasaacH8akY=wiFfYdH8Gipec8Eeeu0xXdbba9frFj0=OqFfea0dXdd9vqai=hGuQ8kuc9pgc9s8qqaq=dirpe0xb9q8qiLsFr0=vr0=vr0dc8meaabaqaciaacaGaaeqabaqabeGadaaakeaacqWGnbqtcqWGtbWucqWGxbWvcqGH9aqpdaWcaaqaaiabigdaXaqaaiabdUgaRjabcIcaOiabd6gaUjabgkHiTiabigdaXiabcMcaPaaadaaeWbqaamaaqahabaWaaeWaaeaacqWG5bqEdaWgaaWcbaGaemyAaKMaemOAaOgabeaakiabgkHiTiqbdMha5zaaraWaaSbaaSqaaiabdMgaPbqabaaakiaawIcacaGLPaaadaahaaWcbeqaaiabikdaYaaaaeaacqWGQbGAaeaacqWGUbGBa0GaeyyeIuoaaSqaaiabdMgaPbqaaiabdUgaRbqdcqGHris5aaaa@4DA5@, MSB=nk−1∑ik(y¯i−μ^)2
 MathType@MTEF@5@5@+=feaafiart1ev1aaatCvAUfKttLearuWrP9MDH5MBPbIqV92AaeXatLxBI9gBaebbnrfifHhDYfgasaacH8akY=wiFfYdH8Gipec8Eeeu0xXdbba9frFj0=OqFfea0dXdd9vqai=hGuQ8kuc9pgc9s8qqaq=dirpe0xb9q8qiLsFr0=vr0=vr0dc8meaabaqaciaacaGaaeqabaqabeGadaaakeaacqWGnbqtcqWGtbWucqWGcbGqcqGH9aqpdaWcaaqaaiabd6gaUbqaaiabdUgaRjabgkHiTiabigdaXaaadaaeWbqaamaabmaabaGafmyEaKNbaebadaWgaaWcbaGaemyAaKgabeaakiabgkHiTGGaciqb=X7aTzaajaaacaGLOaGaayzkaaWaaWbaaSqabeaacqaIYaGmaaaabaGaemyAaKgabaGaem4AaSganiabggHiLdaaaa@4339@ are respectively, the within-subject and between subjects mean squares as obtained from the usual one-way ANOVA table, and y¯i=1n∑j=1nyij
 MathType@MTEF@5@5@+=feaafiart1ev1aaatCvAUfKttLearuWrP9MDH5MBPbIqV92AaeXatLxBI9gBaebbnrfifHhDYfgasaacH8akY=wiFfYdH8Gipec8Eeeu0xXdbba9frFj0=OqFfea0dXdd9vqai=hGuQ8kuc9pgc9s8qqaq=dirpe0xb9q8qiLsFr0=vr0=vr0dc8meaabaqaciaacaGaaeqabaqabeGadaaakeaacuWG5bqEgaqeamaaBaaaleaacqWGPbqAaeqaaOGaeyypa0ZaaSaaaeaacqaIXaqmaeaacqWGUbGBaaWaaabCaeaacqWG5bqEdaWgaaWcbaGaemyAaKMaemOAaOgabeaaaeaacqWGQbGAcqGH9aqpcqaIXaqmaeaacqWGUbGBa0GaeyyeIuoaaaa@3E89@. Note that the *MSB *does not exist for *k *= 1, which means that to obtain a sensible estimate of *ρ *as an index of reliability, the study should include more than one subject.

## Results

The asymptotic variance-covariance matrix of the MLE's is obtained by inverting Fisher's information matrix. The large sample variance of θ^
 MathType@MTEF@5@5@+=feaafiart1ev1aaatCvAUfKttLearuWrP9MDH5MBPbIqV92AaeXatLxBI9gBaebbnrfifHhDYfgasaacH8akY=wiFfYdH8Gipec8Eeeu0xXdbba9frFj0=OqFfea0dXdd9vqai=hGuQ8kuc9pgc9s8qqaq=dirpe0xb9q8qiLsFr0=vr0=vr0dc8meaabaqaciaacaGaaeqabaqabeGadaaakeaaiiGacuWF4oqCgaqcaaaa@2E79@ can be obtained using delta method (see Kendall vol. 1 [13]) and was shown by Quan and Shih [7] to be:

var⁡(θ^)=A(ρ,n,θ)/k=θ4nk[1+nρ1−ρ]+θ22k(n−1)     (3)
 MathType@MTEF@5@5@+=feaafiart1ev1aaatCvAUfKttLearuWrP9MDH5MBPbIqV92AaeXatLxBI9gBaebbnrfifHhDYfgasaacH8akY=wiFfYdH8Gipec8Eeeu0xXdbba9frFj0=OqFfea0dXdd9vqai=hGuQ8kuc9pgc9s8qqaq=dirpe0xb9q8qiLsFr0=vr0=vr0dc8meaabaqaciaacaGaaeqabaqabeGadaaakeaacyGG2bGDcqGGHbqycqGGYbGCcqGGOaakiiGacuWF4oqCgaqcaiabcMcaPiabg2da9iabdgeabjabcIcaOiab=f8aYjabcYcaSiabd6gaUjabcYcaSiab=H7aXjabcMcaPiabc+caViabdUgaRjabg2da9maalaaabaGae8hUde3aaWbaaSqabeaacqaI0aanaaaakeaacqWGUbGBcqWGRbWAaaWaamWaaeaacqaIXaqmcqGHRaWkcqWGUbGBdaWcaaqaaiab=f8aYbqaaiabigdaXiabgkHiTiab=f8aYbaaaiaawUfacaGLDbaacqGHRaWkdaWcaaqaaiab=H7aXnaaCaaaleqabaGaeGOmaidaaaGcbaGaeGOmaiJaem4AaSMaeiikaGIaemOBa4MaeyOeI0IaeGymaeJaeiykaKcaaiaaxMaacaWLjaWaaeWaaeaacqaIZaWmaiaawIcacaGLPaaaaaa@6106@

To construct an approximate confidence interval on θ^
 MathType@MTEF@5@5@+=feaafiart1ev1aaatCvAUfKttLearuWrP9MDH5MBPbIqV92AaeXatLxBI9gBaebbnrfifHhDYfgasaacH8akY=wiFfYdH8Gipec8Eeeu0xXdbba9frFj0=OqFfea0dXdd9vqai=hGuQ8kuc9pgc9s8qqaq=dirpe0xb9q8qiLsFr0=vr0=vr0dc8meaabaqaciaacaGaaeqabaqabeGadaaakeaaiiGacuWF4oqCgaqcaaaa@2E79@, it is assumed that for large *k*, k
 MathType@MTEF@5@5@+=feaafiart1ev1aaatCvAUfKttLearuWrP9MDH5MBPbIqV92AaeXatLxBI9gBaebbnrfifHhDYfgasaacH8akY=wiFfYdH8Gipec8Eeeu0xXdbba9frFj0=OqFfea0dXdd9vqai=hGuQ8kuc9pgc9s8qqaq=dirpe0xb9q8qiLsFr0=vr0=vr0dc8meaabaqaciaacaGaaeqabaqabeGadaaakeaadaGcaaqaaiabdUgaRbWcbeaaaaa@2E26@(θ^
 MathType@MTEF@5@5@+=feaafiart1ev1aaatCvAUfKttLearuWrP9MDH5MBPbIqV92AaeXatLxBI9gBaebbnrfifHhDYfgasaacH8akY=wiFfYdH8Gipec8Eeeu0xXdbba9frFj0=OqFfea0dXdd9vqai=hGuQ8kuc9pgc9s8qqaq=dirpe0xb9q8qiLsFr0=vr0=vr0dc8meaabaqaciaacaGaaeqabaqabeGadaaakeaaiiGacuWF4oqCgaqcaaaa@2E79@ - *θ*) follows a normal distribution with mean 0 and variance A(*ρ*,*n*,*θ*). An approximate 100(1 - *α*)% confidence interval on *θ *can be given as θ^±Ζα2var⁡(θ^)
 MathType@MTEF@5@5@+=feaafiart1ev1aaatCvAUfKttLearuWrP9MDH5MBPbIqV92AaeXatLxBI9gBaebbnrfifHhDYfgasaacH8akY=wiFfYdH8Gipec8Eeeu0xXdbba9frFj0=OqFfea0dXdd9vqai=hGuQ8kuc9pgc9s8qqaq=dirpe0xb9q8qiLsFr0=vr0=vr0dc8meaabaqaciaacaGaaeqabaqabeGadaaakeaaiiGacuWF4oqCgaqcaiabgglaXkab=z5aAnaaBaaaleaadaWccaqaaiabeg7aHbqaaGGaaiab+jdaYaaaaeqaaOWaaOaaaeaacyGG2bGDcqGGHbqycqGGYbGCcqGGOaakcuWF4oqCgaqcaiabcMcaPaWcbeaaaaa@3C72@, where Z_*α*/2 _is the 100(1 - *α*/2)% cut off point of the standard normal distribution.

Due to the dependence of the variance of θ^
 MathType@MTEF@5@5@+=feaafiart1ev1aaatCvAUfKttLearuWrP9MDH5MBPbIqV92AaeXatLxBI9gBaebbnrfifHhDYfgasaacH8akY=wiFfYdH8Gipec8Eeeu0xXdbba9frFj0=OqFfea0dXdd9vqai=hGuQ8kuc9pgc9s8qqaq=dirpe0xb9q8qiLsFr0=vr0=vr0dc8meaabaqaciaacaGaaeqabaqabeGadaaakeaaiiGacuWF4oqCgaqcaaaa@2E79@ on the true parameter value *θ *itself, we found that the asymptotic coverage deviates from its nominal levels for some values of *θ*. To improve the coverage probability we suggest a variance stabilizing transformation to remove the dependence of var(θ^
 MathType@MTEF@5@5@+=feaafiart1ev1aaatCvAUfKttLearuWrP9MDH5MBPbIqV92AaeXatLxBI9gBaebbnrfifHhDYfgasaacH8akY=wiFfYdH8Gipec8Eeeu0xXdbba9frFj0=OqFfea0dXdd9vqai=hGuQ8kuc9pgc9s8qqaq=dirpe0xb9q8qiLsFr0=vr0=vr0dc8meaabaqaciaacaGaaeqabaqabeGadaaakeaaiiGacuWF4oqCgaqcaaaa@2E79@) on *θ*.

### Variance Stabilizing Transformation (VST)

To improve the estimated coverage proportion, we propose a variance stabilizing transformation g (see Kendall vol.1 page 541 [13]) where, g = ∫(var(θ^
 MathType@MTEF@5@5@+=feaafiart1ev1aaatCvAUfKttLearuWrP9MDH5MBPbIqV92AaeXatLxBI9gBaebbnrfifHhDYfgasaacH8akY=wiFfYdH8Gipec8Eeeu0xXdbba9frFj0=OqFfea0dXdd9vqai=hGuQ8kuc9pgc9s8qqaq=dirpe0xb9q8qiLsFr0=vr0=vr0dc8meaabaqaciaacaGaaeqabaqabeGadaaakeaaiiGacuWF4oqCgaqcaaaa@2E79@))^-1/2 ^*dθ*. With *θ *defined as in equation (2), it can be shown that g(θ)=k(n−1)/2log⁡[(1+cθ2)1/2−1(1+cθ2)1/2+1]
 MathType@MTEF@5@5@+=feaafiart1ev1aaatCvAUfKttLearuWrP9MDH5MBPbIqV92AaeXatLxBI9gBaebbnrfifHhDYfgasaacH8akY=wiFfYdH8Gipec8Eeeu0xXdbba9frFj0=OqFfea0dXdd9vqai=hGuQ8kuc9pgc9s8qqaq=dirpe0xb9q8qiLsFr0=vr0=vr0dc8meaabaqaciaacaGaaeqabaqabeGadaaakeaacqWGNbWzcqGGOaakiiGacqWF4oqCcqGGPaqkcqGH9aqpdaGcaaqaaiabdUgaRjabcIcaOiabd6gaUjabgkHiTiabigdaXiabcMcaPiabc+caViabikdaYaWcbeaakiGbcYgaSjabc+gaVjabcEgaNjabcUfaBnaalaaabaWaaeWaaeaacqaIXaqmcqGHRaWkcqWGJbWycqWF4oqCdaahaaWcbeqaaiabikdaYaaaaOGaayjkaiaawMcaamaaCaaaleqabaGaeGymaeJaei4la8IaeGOmaidaaOGaeyOeI0IaeGymaedabaWaaeWaaeaacqaIXaqmcqGHRaWkcqWGJbWycqWF4oqCdaahaaWcbeqaaiabikdaYaaaaOGaayjkaiaawMcaamaaCaaaleqabaGaeGymaeJaei4la8IaeGOmaidaaOGaey4kaSIaeGymaedaaiabc2faDbaa@5A2B@ where, c=2(1−1n)(1+nρ*)
 MathType@MTEF@5@5@+=feaafiart1ev1aaatCvAUfKttLearuWrP9MDH5MBPbIqV92AaeXatLxBI9gBaebbnrfifHhDYfgasaacH8akY=wiFfYdH8Gipec8Eeeu0xXdbba9frFj0=OqFfea0dXdd9vqai=hGuQ8kuc9pgc9s8qqaq=dirpe0xb9q8qiLsFr0=vr0=vr0dc8meaabaqaciaacaGaaeqabaqabeGadaaakeaacqWGJbWycqGH9aqpcqaIYaGmcqGGOaakcqaIXaqmcqGHsisldaWcaaqaaiabigdaXaqaaiabd6gaUbaacqGGPaqkcqGGOaakcqaIXaqmcqGHRaWkcqWGUbGBiiGacqWFbpGCcqGGQaGkcqGGPaqkaaa@3D73@, and ρ* = *ρ*/(1 - *ρ*) Letting f(θ,n,ρ)=(n−1)/2log⁡[(1+cθ2)1/2−1(1+cθ2)1/2+1]
 MathType@MTEF@5@5@+=feaafiart1ev1aaatCvAUfKttLearuWrP9MDH5MBPbIqV92AaeXatLxBI9gBaebbnrfifHhDYfgasaacH8akY=wiFfYdH8Gipec8Eeeu0xXdbba9frFj0=OqFfea0dXdd9vqai=hGuQ8kuc9pgc9s8qqaq=dirpe0xb9q8qiLsFr0=vr0=vr0dc8meaabaqaciaacaGaaeqabaqabeGadaaakeaacqWGMbGzcqGGOaakiiGacqWF4oqCcqGGSaalcqWGUbGBcqGGSaalcqWFbpGCcqGGPaqkcqGH9aqpdaGcaaqaamaabmaabaGaemOBa4MaeyOeI0IaeGymaedacaGLOaGaayzkaaGaei4la8IaeGOmaidaleqaaOGagiiBaWMaei4Ba8Maei4zaCMaei4waS1aaSaaaeaadaqadaqaaiabigdaXiabgUcaRiabdogaJjab=H7aXnaaCaaaleqabaGaeGOmaidaaaGccaGLOaGaayzkaaWaaWbaaSqabeaacqaIXaqmcqGGVaWlcqaIYaGmaaGccqGHsislcqaIXaqmaeaadaqadaqaaiabigdaXiabgUcaRiabdogaJjab=H7aXnaaCaaaleqabaGaeGOmaidaaaGccaGLOaGaayzkaaWaaWbaaSqabeaacqaIXaqmcqGGVaWlcqaIYaGmaaGccqGHRaWkcqaIXaqmaaGaeiyxa0faaa@5D81@, we may establish assuming the function *g *is bounded and differentiable, that, *f*(θ^
 MathType@MTEF@5@5@+=feaafiart1ev1aaatCvAUfKttLearuWrP9MDH5MBPbIqV92AaeXatLxBI9gBaebbnrfifHhDYfgasaacH8akY=wiFfYdH8Gipec8Eeeu0xXdbba9frFj0=OqFfea0dXdd9vqai=hGuQ8kuc9pgc9s8qqaq=dirpe0xb9q8qiLsFr0=vr0=vr0dc8meaabaqaciaacaGaaeqabaqabeGadaaakeaaiiGacuWF4oqCgaqcaaaa@2E79@,*n*,*ρ*) is asymptotically normally distributed with mean *f*(*θ*,*n*,*ρ*) and variance 1/*k*. Therefore, we can construct 100(1- *α*)% confidence limits on *θ *based on the above transformation. The upper and lower (1- *α*/2)100% confidence bound on *θ *are respectively given by:

θ^u=2exp⁡(ξ1(2(n−1))−1/2)c1/2[1−exp⁡(ξ1(2n−1)1/2)]
 MathType@MTEF@5@5@+=feaafiart1ev1aaatCvAUfKttLearuWrP9MDH5MBPbIqV92AaeXatLxBI9gBaebbnrfifHhDYfgasaacH8akY=wiFfYdH8Gipec8Eeeu0xXdbba9frFj0=OqFfea0dXdd9vqai=hGuQ8kuc9pgc9s8qqaq=dirpe0xb9q8qiLsFr0=vr0=vr0dc8meaabaqaciaacaGaaeqabaqabeGadaaakeaaiiGacuWF4oqCgaqcamaaBaaaleaacqWG1bqDaeqaaOGaeyypa0ZaaSaaaeaacqaIYaGmcyGGLbqzcqGG4baEcqGGWbaCcqGGOaakcqWF+oaEdaWgaaWcbaGaeGymaedabeaakiabcIcaOiabikdaYiabcIcaOiabd6gaUjabgkHiTiabigdaXiabcMcaPiabcMcaPmaaCaaaleqabaGaeyOeI0IaeGymaeJaei4la8IaeGOmaidaaOGaeiykaKcabaGaem4yam2aaWbaaSqabeaacqaIXaqmcqGGVaWlcqaIYaGmaaGccqGGBbWwcqaIXaqmcqGHsislcyGGLbqzcqGG4baEcqGGWbaCcqGGOaakcqWF+oaEdaWgaaWcbaGaeGymaedabeaakiabcIcaOmaalaaabaGaeGOmaidabaGaemOBa4MaeyOeI0IaeGymaedaaiabcMcaPmaaCaaaleqabaGaeGymaeJaei4la8IaeGOmaidaaOGaeiykaKIaeiyxa0faaaaa@60E2@ and,

θ^l=2exp⁡(ξ2(2(n−1))−1/2)c1/2[1−exp⁡(ξ2(2n−1)1/2)]
 MathType@MTEF@5@5@+=feaafiart1ev1aaatCvAUfKttLearuWrP9MDH5MBPbIqV92AaeXatLxBI9gBaebbnrfifHhDYfgasaacH8akY=wiFfYdH8Gipec8Eeeu0xXdbba9frFj0=OqFfea0dXdd9vqai=hGuQ8kuc9pgc9s8qqaq=dirpe0xb9q8qiLsFr0=vr0=vr0dc8meaabaqaciaacaGaaeqabaqabeGadaaakeaaiiGacuWF4oqCgaqcamaaBaaaleaacqWGSbaBaeqaaOGaeyypa0ZaaSaaaeaacqaIYaGmcyGGLbqzcqGG4baEcqGGWbaCcqGGOaakcqWF+oaEdaWgaaWcbaGaeGOmaidabeaakiabcIcaOiabikdaYiabcIcaOiabd6gaUjabgkHiTiabigdaXiabcMcaPiabcMcaPmaaCaaaleqabaGaeyOeI0IaeGymaeJaei4la8IaeGOmaidaaOGaeiykaKcabaGaem4yam2aaWbaaSqabeaacqaIXaqmcqGGVaWlcqaIYaGmaaGccqGGBbWwcqaIXaqmcqGHsislcyGGLbqzcqGG4baEcqGGWbaCcqGGOaakcqWF+oaEdaWgaaWcbaGaeGOmaidabeaakiabcIcaOmaalaaabaGaeGOmaidabaGaemOBa4MaeyOeI0IaeGymaedaaiabcMcaPmaaCaaaleqabaGaeGymaeJaei4la8IaeGOmaidaaOGaeiykaKIaeiyxa0faaaaa@60D4@

where, *ξ*_1 _= *f*(θ^
 MathType@MTEF@5@5@+=feaafiart1ev1aaatCvAUfKttLearuWrP9MDH5MBPbIqV92AaeXatLxBI9gBaebbnrfifHhDYfgasaacH8akY=wiFfYdH8Gipec8Eeeu0xXdbba9frFj0=OqFfea0dXdd9vqai=hGuQ8kuc9pgc9s8qqaq=dirpe0xb9q8qiLsFr0=vr0=vr0dc8meaabaqaciaacaGaaeqabaqabeGadaaakeaaiiGacuWF4oqCgaqcaaaa@2E79@,*n*,*ρ*) + *z*_*α*/2_/k
 MathType@MTEF@5@5@+=feaafiart1ev1aaatCvAUfKttLearuWrP9MDH5MBPbIqV92AaeXatLxBI9gBaebbnrfifHhDYfgasaacH8akY=wiFfYdH8Gipec8Eeeu0xXdbba9frFj0=OqFfea0dXdd9vqai=hGuQ8kuc9pgc9s8qqaq=dirpe0xb9q8qiLsFr0=vr0=vr0dc8meaabaqaciaacaGaaeqabaqabeGadaaakeaadaGcaaqaaiabdUgaRbWcbeaaaaa@2E26@, and *ξ*_2 _= *f*(θ^
 MathType@MTEF@5@5@+=feaafiart1ev1aaatCvAUfKttLearuWrP9MDH5MBPbIqV92AaeXatLxBI9gBaebbnrfifHhDYfgasaacH8akY=wiFfYdH8Gipec8Eeeu0xXdbba9frFj0=OqFfea0dXdd9vqai=hGuQ8kuc9pgc9s8qqaq=dirpe0xb9q8qiLsFr0=vr0=vr0dc8meaabaqaciaacaGaaeqabaqabeGadaaakeaaiiGacuWF4oqCgaqcaaaa@2E79@,*n*,*ρ*) - *z*_*α*/2_/k
 MathType@MTEF@5@5@+=feaafiart1ev1aaatCvAUfKttLearuWrP9MDH5MBPbIqV92AaeXatLxBI9gBaebbnrfifHhDYfgasaacH8akY=wiFfYdH8Gipec8Eeeu0xXdbba9frFj0=OqFfea0dXdd9vqai=hGuQ8kuc9pgc9s8qqaq=dirpe0xb9q8qiLsFr0=vr0=vr0dc8meaabaqaciaacaGaaeqabaqabeGadaaakeaadaGcaaqaaiabdUgaRbWcbeaaaaa@2E26@.

Note that the limits of the interval depend on the unknown value of the intra-class correlation, which can be replaced by its MLE as defined in section 2.1.

To examine the finite sample behavior of the VST based confidence interval estimator, a Monte-Carlo study was conducted under model (1) using the S-Plus program. The values of *ρ *were, 0.3, 0.4, 0.6, 0.7, and 0.8; *μ *= 10, and *θ *= 4%, 10% or 20%. Sample size (*k*) = 12, 25, 50, 75, and number of replicates (*n*) = 2, 3, and 5. The number of repetitions for each simulation was 1000. Tables 1, 2, 3, and 4, demonstrate the coverage proportion for the 95% nominal level confidence interval on the WCV. The estimated coverage proportions were close to the 95% nominal level.

**Table 1:** Estimated coverage probabilities under the VST. The nominal level is 95%. (*θ *= 0.04)

*k*	*ρ*	*n*		
		
		2	3	5
	0.3	0.929	0.934	0.947
	0.4	0.943	0.937	0.930
12	0.6	0.943	0.948	0.938
	0.7	0.941	0.943	0.934
	0.8	0.939	0.931	0.941
	0.3	0.961	0.946	0.949
	0.4	0.942	0.946	0.953
25	0.6	0.939	0.935	0.969
	0.7	0.946	0.936	0.948
	0.8	0.936	0.936	0.940
	0.3	0.956	0.954	0.949
	0.4	0.945	0.939	0.950
50	0.6	0.952	0.955	0.934
	0.7	0.953	0.948	0.940
	0.8	0.950	0.935	0.937
	0.3	0.948	0.948	0.944
	0.4	0.956	0.955	0.955
75	0.6	0.952	0.943	0.949
	0.7	0.945	0.944	0.930
	0.8	0.946	0.945	0.946

**Table 2:** Estimated coverage probabilities under the VST. The nominal level is 95%. (*θ *= 0.01)

*K*	*ρ*	*n*		
		
		2	3	5
	0.3	0.943	0.954	0.945
	0.4	0.930	0.934	0.952
12	0.6	0.949	0.942	0.938
	0.7	0.920	0.932	0.929
	0.8	0.927	0.926	0.913
	0.3	0.938	0.951	0.945
	0.4	0.936	0.953	0.955
25	0.6	0.926	0.948	0.946
	0.7	0.928	0.939	0.931
	0.8	0.925	0.939	0.915
	0.3	0.946	0.936	0.946
	0.4	0.957	0.935	0.942
50	0.6	0.940	0.955	0.936
	0.7	0.940	0.947	0.935
	0.8	0.947	0.930	0.925
	0.3	0.941	0.948	0.945
	0.4	0.941	0.947	0.941
75	0.6	0.935	0.933	0.941
	0.7	0.932	0.929	0.949
	0.8	0.960	0.936	0.925

**Table 3:** Estimated coverage probabilities under the VST. The nominal level is 95%. (*θ *= 0.02)

*K*	*ρ*	*n*		
		
		2	3	5
	0.3	0.947	0.941	0.945
	0.4	0.936	0.945	0.942
12	0.6	0.932	0.939	0.950
	0.7	0.937	0.938	0.935
	0.8	0.934	0.925	0.921
	0.3	0.951	0.951	0.954
	0.4	0.942	0.943	0.943
25	0.6	0.940	0.946	0.934
	0.7	0.940	0.941	0.920
	0.8	0.939	0.934	0.909
	0.3	0.948	0.947	0.935
	0.4	0.941	0.945	0.948
50	0.6	0.939	0.952	0.947
	0.7	0.939	0.944	0.949
	0.8	0.941	0.931	0.908
	0.3	0.951	0.953	0.956
	0.4	0.948	0.947	0.951
75	0.6	0.950	0.945	0.944
	0.7	0.936	0.931	0.931
	0.8	0.937	0.930	0.912

**Table 4:** Results for 10 replicates on each of three patient's total lesion burden. Values are given volumes in cubic centimeters.

Replicates											
Patient	Method*	1	2	3	4	5	6	7	8	9	10

1	M	20	21.2	20.8	20.6	20.2	19.1	21	20.4	19.2	19.2
	G	19.5	19.5	19.6	19.7	19.3	19.1	19.1	19.3	19.2	19.5
2	M	26.8	26.5	22.5	23.1	24.3	24.1	26	26.8	24.9	27.7
	G	22.1	21.9	22	22.1	21.9	21.8	21.7	21.7	21.7	21.8
3	M	9.6	10.5	10.6	9.2	10.4	10.4	10.1	8	10.1	8.9
	G	8.5	8.5	8.3	8.3	8.3	8	8	8	8	8.1

*M = Manual, G = Geometrically constrained region growth.

### Example 1

Accurate and reproducible quantification of brain lesion count and volume in multiple sclerosis (MS) patients using magnetic resonance imaging (MRI) is a vital tool for evaluation of disease progression and patient response to therapy. Current standard methods for obtaining these data are largely manual and subjective and are therefore error-prone and subject to inter-and intra-operator variability. Therefore, there is a need for a rapid automated lesion quantification method. Ashton *et al*. [1] compared manual measurements and an automated data technique known as Geometrically Constrained Region Growth (GEORG) of the brain lesion volume of 3 MS patients, each measured 10 times by a single operator for each method. The data are presented in Table 5.

**Table 5:** Summary analysis of data in Table 2 and 95% confidence intervals

Method	ρ^ MathType@MTEF@5@5@+=feaafiart1ev1aaatCvAUfKttLearuWrP9MDH5MBPbIqV92AaeXatLxBI9gBaebbnrfifHhDYfgasaacH8akY=wiFfYdH8Gipec8Eeeu0xXdbba9frFj0=OqFfea0dXdd9vqai=hGuQ8kuc9pgc9s8qqaq=dirpe0xb9q8qiLsFr0=vr0=vr0dc8meaabaqaciaacaGaaeqabaqabeGadaaakeaaiiGacuWFbpGCgaqcaaaa@2E83@	θ^ MathType@MTEF@5@5@+=feaafiart1ev1aaatCvAUfKttLearuWrP9MDH5MBPbIqV92AaeXatLxBI9gBaebbnrfifHhDYfgasaacH8akY=wiFfYdH8Gipec8Eeeu0xXdbba9frFj0=OqFfea0dXdd9vqai=hGuQ8kuc9pgc9s8qqaq=dirpe0xb9q8qiLsFr0=vr0=vr0dc8meaabaqaciaacaGaaeqabaqabeGadaaakeaaiiGacuWF4oqCgaqcaaaa@2E79@	95% CI without VST	95% CI with VST
M	0.966	6.5%	(0.034, 0.096)	(0.043, 0.118)
G	0.999	1.2%	(0.006, 0.017)	(0.008, 0.021)

Based on the guidelines for the levels of reliability provided by Fleiss [4], a value of an ICC above 80% indicates an excellent reliability, and from Table 3 both methods cross this threshold level. However, based on the WSCV values, the manual method is definitely less reproducible than the automated method (the GEORG is 5 times more reproducible than the manual). This example demonstrates the usefulness of the WSCV over the ICC as a measure of reproducibility. Clearly, one should construct a formal test on the significance of the difference between two correlated within-subject coefficients of variation. There are several competing methods to construct such a test (e.g. LRT, Wald, and Score tests) but this issue is quite involved and so we intend to report our findings in a future publication.

### Sample size estimation

In the following development we discuss the second objective of this paper. We assume that the investigator is interested in the number of replicates, *n*, per subject, so that the variance of the estimate of *θ *is minimized, given that the total number of measurements is fixed *a priori *at *N *= *nk*.

### Efficiency criterion

For fixed total number of measurements *N *= *nk*, equation (3) gives:

var⁡(θ^)=θ4N[1+nρ1−ρ]+nθ22N(n−1)     (4)
 MathType@MTEF@5@5@+=feaafiart1ev1aaatCvAUfKttLearuWrP9MDH5MBPbIqV92AaeXatLxBI9gBaebbnrfifHhDYfgasaacH8akY=wiFfYdH8Gipec8Eeeu0xXdbba9frFj0=OqFfea0dXdd9vqai=hGuQ8kuc9pgc9s8qqaq=dirpe0xb9q8qiLsFr0=vr0=vr0dc8meaabaqaciaacaGaaeqabaqabeGadaaakeaacyGG2bGDcqGGHbqycqGGYbGCcqGGOaakiiGacuWF4oqCgaqcaiabcMcaPiabg2da9maalaaabaGae8hUde3aaWbaaSqabeaacqaI0aanaaaakeaacqWGobGtaaWaamWaaeaacqaIXaqmcqGHRaWkcqWGUbGBdaWcaaqaaiab=f8aYbqaaiabigdaXiabgkHiTiab=f8aYbaaaiaawUfacaGLDbaacqGHRaWkdaWcaaqaaiabd6gaUjab=H7aXnaaCaaaleqabaGaeGOmaidaaaGcbaGaeGOmaiJaemOta4KaeiikaGIaemOBa4MaeyOeI0IaeGymaeJaeiykaKcaaiaaxMaacaWLjaWaaeWaaeaacqaI0aanaiaawIcacaGLPaaaaaa@53FB@

The necessary condition for var(θ^
 MathType@MTEF@5@5@+=feaafiart1ev1aaatCvAUfKttLearuWrP9MDH5MBPbIqV92AaeXatLxBI9gBaebbnrfifHhDYfgasaacH8akY=wiFfYdH8Gipec8Eeeu0xXdbba9frFj0=OqFfea0dXdd9vqai=hGuQ8kuc9pgc9s8qqaq=dirpe0xb9q8qiLsFr0=vr0=vr0dc8meaabaqaciaacaGaaeqabaqabeGadaaakeaaiiGacuWF4oqCgaqcaaaa@2E79@) to have a unique minimum is that ∂var(θ^
 MathType@MTEF@5@5@+=feaafiart1ev1aaatCvAUfKttLearuWrP9MDH5MBPbIqV92AaeXatLxBI9gBaebbnrfifHhDYfgasaacH8akY=wiFfYdH8Gipec8Eeeu0xXdbba9frFj0=OqFfea0dXdd9vqai=hGuQ8kuc9pgc9s8qqaq=dirpe0xb9q8qiLsFr0=vr0=vr0dc8meaabaqaciaacaGaaeqabaqabeGadaaakeaaiiGacuWF4oqCgaqcaaaa@2E79@)/∂*n *= 0. This, and the additional condition that ∂^2 ^var(θ^
 MathType@MTEF@5@5@+=feaafiart1ev1aaatCvAUfKttLearuWrP9MDH5MBPbIqV92AaeXatLxBI9gBaebbnrfifHhDYfgasaacH8akY=wiFfYdH8Gipec8Eeeu0xXdbba9frFj0=OqFfea0dXdd9vqai=hGuQ8kuc9pgc9s8qqaq=dirpe0xb9q8qiLsFr0=vr0=vr0dc8meaabaqaciaacaGaaeqabaqabeGadaaakeaaiiGacuWF4oqCgaqcaaaa@2E79@)/∂*n*^2 ^> 0 are both satisfied so long as 0 <*ρ *< 1. Differentiating (4) with respect to *n*, equating to zero and solving for *n *we obtain

n*=1+(1−ρ)2ρθ20<ρ<1.     (5)
 MathType@MTEF@5@5@+=feaafiart1ev1aaatCvAUfKttLearuWrP9MDH5MBPbIqV92AaeXatLxBI9gBaebbnrfifHhDYfgasaacH8akY=wiFfYdH8Gipec8Eeeu0xXdbba9frFj0=OqFfea0dXdd9vqai=hGuQ8kuc9pgc9s8qqaq=dirpe0xb9q8qiLsFr0=vr0=vr0dc8meaabaqaciaacaGaaeqabaqabeGadaaakeaafaqabeqacaaabaGaemOBa4MaeiOkaOIaeyypa0JaeGymaeJaey4kaSYaaOaaaeaadaWcaaqaaiabcIcaOiabigdaXiabgkHiTGGaciab=f8aYjabcMcaPaqaaiabikdaYiab=f8aYjab=H7aXnaaCaaaleqabaGaeGOmaidaaaaaaeqaaaGcbaGaeGimaaJaeyipaWJae8xWdiNaeyipaWJaeGymaeJaeiOla4caaiaaxMaacaWLjaWaaeWaaeaacqaI1aqnaiaawIcacaGLPaaaaaa@4719@

The required number of subject is thus k* = *N*/n*.

Table 4 shows few optimal allocations of (*n*, *k*) for *ρ *= 0.6, 0.7, and 0.8, *θ *= 0.1, 0.2, 0.3 and 0.4, when *N *= 24.

Note that in practice, only integer values of (*n*, *k*) are used, and because *N *= *nk *is fixed *a priori*, we first round the optimum values of *n *to the nearest integer; then *k *= *N/n *was rounded to the nearest integer. The values of var(θ^
 MathType@MTEF@5@5@+=feaafiart1ev1aaatCvAUfKttLearuWrP9MDH5MBPbIqV92AaeXatLxBI9gBaebbnrfifHhDYfgasaacH8akY=wiFfYdH8Gipec8Eeeu0xXdbba9frFj0=OqFfea0dXdd9vqai=hGuQ8kuc9pgc9s8qqaq=dirpe0xb9q8qiLsFr0=vr0=vr0dc8meaabaqaciaacaGaaeqabaqabeGadaaakeaaiiGacuWF4oqCgaqcaaaa@2E79@) at the rounded optimal allocations for different values of *ρ*,*θ *and *n *showed that the net loss or gain in efficiency due to rounding is negligible. It is clear that to efficiently estimate the WSCV for large values of *θ *we need smaller number of replicates and larger number of subjects.

### Fixed width confidence interval approach

Bonett [10] discussed the issue of sample size requirements that achieve a pre-specified expected width for a confidence interval about ICC. This approach is useful in planning a reliability study in which the focus is on estimation rather than hypothesis testing. He demonstrated that the effect of inaccurate planning value of ICC is more serious in hypothesis testing applications. Shoukri et al. [11] argued that the hypothesis testing approach might not be appropriate while planning a reproducibility study. This is because, in most cases, values of the coefficient under the null and alternative hypotheses may be difficult to specify. An alternative approach is to focus on the width of the CI for *θ*. Since the approximate width of an (1 - *α*)100%CI on *θ *is, 2z_*α*/2 _var(θ^
 MathType@MTEF@5@5@+=feaafiart1ev1aaatCvAUfKttLearuWrP9MDH5MBPbIqV92AaeXatLxBI9gBaebbnrfifHhDYfgasaacH8akY=wiFfYdH8Gipec8Eeeu0xXdbba9frFj0=OqFfea0dXdd9vqai=hGuQ8kuc9pgc9s8qqaq=dirpe0xb9q8qiLsFr0=vr0=vr0dc8meaabaqaciaacaGaaeqabaqabeGadaaakeaaiiGacuWF4oqCgaqcaaaa@2E79@)^1/2^, an approximate sample size that yields an (1 - *α*)100% CI for *θ *with a desired width *w *obtains by setting *w *= 2*z*_*α*/2 _{var(*θ*)}^1/2 ^and then solving for *k*:

k=4z2A(ρ,n,θ)w2.
 MathType@MTEF@5@5@+=feaafiart1ev1aaatCvAUfKttLearuWrP9MDH5MBPbIqV92AaeXatLxBI9gBaebbnrfifHhDYfgasaacH8akY=wiFfYdH8Gipec8Eeeu0xXdbba9frFj0=OqFfea0dXdd9vqai=hGuQ8kuc9pgc9s8qqaq=dirpe0xb9q8qiLsFr0=vr0=vr0dc8meaabaqaciaacaGaaeqabaqabeGadaaakeaacqWGRbWAcqGH9aqpdaWcaaqaaiabisda0iabdQha6naaCaaaleqabaGaeGOmaidaaOGaemyqaeKaeiikaGccciGae8xWdiNaeiilaWIaemOBa4MaeiilaWIae8hUdeNaeiykaKcabaGaem4DaC3aaWbaaSqabeaacqaIYaGmaaaaaOGaeiOla4caaa@3F9B@

We observe that, for fixed *n *and *θ*, larger values of *ρ *require larger number of subjects to satisfy the criterion. As an example, suppose that it is of interest to construct 95% CI on *θ *with expected width *w *= 0.05, *ρ *= 0.3, and an afforded number of replicates *n *= 2. If the hypothesized value of *θ *is 0.10, then *k *= 31, and if *θ *is 0.3 (*i.e*. lower reliability), then *k *= 323.

### Cost criterion

Funding constraints will often determine the cost of recruiting subjects for a reliability study. Although too small a sample may lead to a study that produces an imprecise estimate of the reproducibility coefficient, too large a sample may result in a waste of resources. Thus, an important decision in a typical reliability study is to balance the cost of recruiting subjects with the need for a precise estimate of the parameter summarizing reliability.

In this section, we determine the combinations (*n*, *k*) that minimize the variance of θ^
 MathType@MTEF@5@5@+=feaafiart1ev1aaatCvAUfKttLearuWrP9MDH5MBPbIqV92AaeXatLxBI9gBaebbnrfifHhDYfgasaacH8akY=wiFfYdH8Gipec8Eeeu0xXdbba9frFj0=OqFfea0dXdd9vqai=hGuQ8kuc9pgc9s8qqaq=dirpe0xb9q8qiLsFr0=vr0=vr0dc8meaabaqaciaacaGaaeqabaqabeGadaaakeaaiiGacuWF4oqCgaqcaaaa@2E79@ subject to cost constraints. Constructing a flexible cost function starts with identifying sampling and overhead costs. The sampling cost depends primarily on the size of the sample and includes costs for data collection, compensation to volunteers, management, and evaluation. On the other hand, overhead costs are independent of sample size. Following Sukhatme et al. [14], we assume that the overall cost function is given as:

*C *= *c*_0 _+ *kc*_1 _+ *nkc*_2 _    (6)

where, *c*_0 _is the fixed cost, *c*_1 _the cost of recruiting a single subject, and *c*_2_is the cost of making one observation. Using the method of Lagrange multipliers and following Shoukri et al. [11], we write the objective function *Ψ *in this form

*Ψ *= var(θ^
 MathType@MTEF@5@5@+=feaafiart1ev1aaatCvAUfKttLearuWrP9MDH5MBPbIqV92AaeXatLxBI9gBaebbnrfifHhDYfgasaacH8akY=wiFfYdH8Gipec8Eeeu0xXdbba9frFj0=OqFfea0dXdd9vqai=hGuQ8kuc9pgc9s8qqaq=dirpe0xb9q8qiLsFr0=vr0=vr0dc8meaabaqaciaacaGaaeqabaqabeGadaaakeaaiiGacuWF4oqCgaqcaaaa@2E79@) + *λ*(*C *- *c*_0 _- *kc*_1 _- *nkc*_2_)     (7)

where, var(θ^
 MathType@MTEF@5@5@+=feaafiart1ev1aaatCvAUfKttLearuWrP9MDH5MBPbIqV92AaeXatLxBI9gBaebbnrfifHhDYfgasaacH8akY=wiFfYdH8Gipec8Eeeu0xXdbba9frFj0=OqFfea0dXdd9vqai=hGuQ8kuc9pgc9s8qqaq=dirpe0xb9q8qiLsFr0=vr0=vr0dc8meaabaqaciaacaGaaeqabaqabeGadaaakeaaiiGacuWF4oqCgaqcaaaa@2E79@) is given by Equation (3) and *λ *is the Lagrange multiplier. Differentiating *Ψ *with respect to *n*, *k *and *λ *and equating to zero, we obtain

2*θ*^2 ^ρ* *n*^4 ^- 4*θ*^2 ^ρ* *n*^3 ^-(2*θ*^2^*r *+ *r *- 2*θ*^2 ^ρ* + 1)*n*^2 ^+ 4*θ*^2^*rn *- 2*θ*^2^*r *= 0     (8)

where *r *= *c*_1_/*c*_2_, and ρ* = *ρ*/(1 - *ρ*)

Although an explicit solution to (8) is available, the resulting expression is complicated and does not provide any useful insight. The 4^*th *^degree polynomial in the left side of (8) has two imaginary roots, one negative and one admissible (positive) root for *n*. Table 5 summarize the results of the optimization procedure where we provide the optimal *n *for various values of *θ*, *ρ*, and *r*, noting that:

kopt=(C−c0)/c2r+nopt.     (9)
 MathType@MTEF@5@5@+=feaafiart1ev1aaatCvAUfKttLearuWrP9MDH5MBPbIqV92AaeXatLxBI9gBaebbnrfifHhDYfgasaacH8akY=wiFfYdH8Gipec8Eeeu0xXdbba9frFj0=OqFfea0dXdd9vqai=hGuQ8kuc9pgc9s8qqaq=dirpe0xb9q8qiLsFr0=vr0=vr0dc8meaabaqaciaacaGaaeqabaqabeGadaaakeaacqWGRbWAdaWgaaWcbaGaem4Ba8MaemiCaaNaemiDaqhabeaakiabg2da9maalaaabaGaeiikaGIaem4qamKaeyOeI0Iaem4yam2aaSbaaSqaaiabicdaWaqabaGccqGGPaqkcqGGVaWlcqWGJbWydaWgaaWcbaGaeGOmaidabeaaaOqaaiabdkhaYjabgUcaRiabd6gaUnaaBaaaleaacqWGVbWBcqWGWbaCcqWG0baDaeqaaaaakiabc6caUiaaxMaacaWLjaWaaeWaaeaacqaI5aqoaiaawIcacaGLPaaaaaa@49F2@

## Results

From Table 7, it is apparent that when *r *= *c*_1_/*c*_2 _increases, the required number of replicates per subject (*n*) increases, because the cost of making a single observation (*c*_2_) decreases and the cost of recruiting a subject (*c*_1_) increases. When *r *is fixed, an increase in *ρ *results in a decline in the required value of *n *and accordingly an increase in *k*. An increase in *θ *also results in a decrease in *n*. The general conclusion is that it is sensible to decrease the number of items associated with a higher cost, while increasing those with lower cost.

**Table 6:** Optimal combinations of (*n*_*opt*_, *k*_*opt*_) which minimize the variance of θ^
 MathType@MTEF@5@5@+=feaafiart1ev1aaatCvAUfKttLearuWrP9MDH5MBPbIqV92AaeXatLxBI9gBaebbnrfifHhDYfgasaacH8akY=wiFfYdH8Gipec8Eeeu0xXdbba9frFj0=OqFfea0dXdd9vqai=hGuQ8kuc9pgc9s8qqaq=dirpe0xb9q8qiLsFr0=vr0=vr0dc8meaabaqaciaacaGaaeqabaqabeGadaaakeaaiiGacuWF4oqCgaqcaaaa@2E79@ for N = 24.

θ	ρ		
	
	0.6	0.7	0.8
	
	(*n*_*opt*_, *k*_*opt*_)		
0.1	(6.77, 3.54)	(5.63, 4.26)	(4.54, 5.29)
0.2	(3.89, 6.17)	(3.31, 7.24)	(2.77, 8.67)
0.3	(2.91, 8.23)	(2.54, 9.47)	(2.17, 11.05)
0.4	(2.44, 9.82)	(2.16, 11.12)	(1.88, 12.74)

**Table 7:** Optimal replications (rounded to the nearest integer) of *n *that minimize the variance of θ^
 MathType@MTEF@5@5@+=feaafiart1ev1aaatCvAUfKttLearuWrP9MDH5MBPbIqV92AaeXatLxBI9gBaebbnrfifHhDYfgasaacH8akY=wiFfYdH8Gipec8Eeeu0xXdbba9frFj0=OqFfea0dXdd9vqai=hGuQ8kuc9pgc9s8qqaq=dirpe0xb9q8qiLsFr0=vr0=vr0dc8meaabaqaciaacaGaaeqabaqabeGadaaakeaaiiGacuWF4oqCgaqcaaaa@2E79@ subject to cost constraints.

				***r***						
				
				**0.1**	**0.5**	**1**	**1.5**	**5**	**10**	**20**
				***n***_***opt***_						
				
			**0.6**	62	72	83	92	142	193	266
	**0.01**		**0.7**	50	58	66	74	114	155	213
			**0.8**	38	44	52	57	88	118	163
			**0.6**	16	19	21	24	36	49	67
	**0.04**		**0.7**	13	15	17	19	29	39	54
			**0.8**	10	12	14	15	23	30	42
			**0.6**	7	8	9	10	15	20	28
	**0.10**		**0.7**	6	7	8	8	12	17	22
			**0.8**	5	5	6	7	10	13	17
**θ**		**ρ**								
			**0.6**	5	6	7	7	11	14	19
	**0.15**		**0.7**	4	5	5	6	9	11	15
			**0.8**	3	4	4	5	7	9	12
			**0.6**	3	3	4	4	6	8	10
	**0.30**		**0.7**	3	3	3	4	5	6	9
			**0.8**	2	2	3	3	4	5	8
			**0.6**	3	3	3	3	5	6	8
	**0.40**		**0.7**	2	2	3	3	4	5	7
			**0.8**	2	2	2	2	3	4	5

We note that by setting *c*_1 _= 0 in Equation (8), we obtain nopt=1+(1−ρ)/2ρθ2
 MathType@MTEF@5@5@+=feaafiart1ev1aaatCvAUfKttLearuWrP9MDH5MBPbIqV92AaeXatLxBI9gBaebbnrfifHhDYfgasaacH8akY=wiFfYdH8Gipec8Eeeu0xXdbba9frFj0=OqFfea0dXdd9vqai=hGuQ8kuc9pgc9s8qqaq=dirpe0xb9q8qiLsFr0=vr0=vr0dc8meaabaqaciaacaGaaeqabaqabeGadaaakeaacqWGUbGBdaWgaaWcbaGaem4Ba8MaemiCaaNaemiDaqhabeaakiabg2da9iabigdaXiabgUcaRmaakaaabaGaeiikaGIaeGymaeJaeyOeI0ccciGae8xWdiNaeiykaKIaei4la8IaeGOmaiJae8xWdiNae8hUde3aaWbaaSqabeaacqaIYaGmaaaabeaaaaa@4129@, as in Equation (5). The situation *c*_1 _= 0 is quite plausible, at least approximately if the major cost is in actually making the observations (e.g. expensive equipment, cost of interviews versus free volunteer subjects). This means that a special cost structure is implied by the optimal allocation procedure discussed earlier.

### Example 2

To assess the accuracy of Doppler Echocardiography (DE) in determining aortic valve area (AVA) prospective evaluation on patients with aortic stenosis, an investigator wishes to demonstrate a high degree of reliability *(ρ *= 0.80) in estimating AVA using the "velocity integral method" with a planned value for the WSCV = 0.10. Suppose that the total cost of making the study is fixed at $1600.0. It is assumed that the overhead fixed cost *c*_0 _is absorbed by the hospital. Moreover, we assume that travel cost is $200.0, and the administrative cost using the DE is $200.0 per visit. From Table 5, *n*_*opt*_for *r *= 1, *ρ *= 0.8, and *θ *= 0.10 is 6. From (9), *k*_*opt *_= (1600/15)/(1 + 6) = 15. That is we need 15 patients, with 6 measurements each to minimize var(θ^
 MathType@MTEF@5@5@+=feaafiart1ev1aaatCvAUfKttLearuWrP9MDH5MBPbIqV92AaeXatLxBI9gBaebbnrfifHhDYfgasaacH8akY=wiFfYdH8Gipec8Eeeu0xXdbba9frFj0=OqFfea0dXdd9vqai=hGuQ8kuc9pgc9s8qqaq=dirpe0xb9q8qiLsFr0=vr0=vr0dc8meaabaqaciaacaGaaeqabaqabeGadaaakeaaiiGacuWF4oqCgaqcaaaa@2E79@) subject to the given cost.

### Estimating the WSCV for dichotomous responses

#### Assumptions

Consider a random sample of *k *subjects, each is blindly evaluated *n *times by the same rater. We assume that all subject responses *y*_*ij *_(where *j *= 1, 2, ...*n*) are dichotomous and are conditionally independent with probabilities *P*(*y*_*ij *_= 1) = *p*_*i *_(*i *= 1,2,...,*k*) and *p*(*y*_*ij *_= 0) = 1 - *p*_*i*_. Thus, for fixed *p*_*i*_, the conditional distribution of the random variable yi·=∑jyij
 MathType@MTEF@5@5@+=feaafiart1ev1aaatCvAUfKttLearuWrP9MDH5MBPbIqV92AaeXatLxBI9gBaebbnrfifHhDYfgasaacH8akY=wiFfYdH8Gipec8Eeeu0xXdbba9frFj0=OqFfea0dXdd9vqai=hGuQ8kuc9pgc9s8qqaq=dirpe0xb9q8qiLsFr0=vr0=vr0dc8meaabaqaciaacaGaaeqabaqabeGadaaakeaacqWG5bqEdaWgaaWcbaGaemyAaKMaeS4JPFgabeaakiabg2da9maaqafabaGaemyEaK3aaSbaaSqaaiabdMgaPjabdQgaQbqabaaabaGaemOAaOgabeqdcqGHris5aaaa@3B02@ follows binomial distribution with parameters *n *and *p*_*i*_. To account for the variation of response probabilities between subjects, as considered by Mak [15], we assume further that the probabilities *p*_*i *_are independently and identically distributed as a beta distribution, Beta (*α*,*β*), with mean *π *= *α*/(*α *+ *β*) and variance *π *(1 - *π*)*ρ*. Given these assumptions, one can show that the correlation between *y*_*ij *_and *y*_*il *_is in fact *ρ*. Define y¯
 MathType@MTEF@5@5@+=feaafiart1ev1aaatCvAUfKttLearuWrP9MDH5MBPbIqV92AaeXatLxBI9gBaebbnrfifHhDYfgasaacH8akY=wiFfYdH8Gipec8Eeeu0xXdbba9frFj0=OqFfea0dXdd9vqai=hGuQ8kuc9pgc9s8qqaq=dirpe0xb9q8qiLsFr0=vr0=vr0dc8meaabaqaciaacaGaaeqabaqabeGadaaakeaacuWG5bqEgaqeaaaa@2E3F@_*i*• _= *y*_*i*•_/*n *and si2=∑j(yij−y¯i·)2
 MathType@MTEF@5@5@+=feaafiart1ev1aaatCvAUfKttLearuWrP9MDH5MBPbIqV92AaeXatLxBI9gBaebbnrfifHhDYfgasaacH8akY=wiFfYdH8Gipec8Eeeu0xXdbba9frFj0=OqFfea0dXdd9vqai=hGuQ8kuc9pgc9s8qqaq=dirpe0xb9q8qiLsFr0=vr0=vr0dc8meaabaqaciaacaGaaeqabaqabeGadaaakeaacqWGZbWCdaqhaaWcbaGaemyAaKgabaGaeGOmaidaaOGaeyypa0ZaaabuaeaadaqadaqaaiabdMha5naaBaaaleaacqWGPbqAcqWGQbGAaeqaaOGaeyOeI0IafmyEaKNbaebadaWgaaWcbaGaemyAaKMaeS4JPFgabeaaaOGaayjkaiaawMcaaaWcbaGaemOAaOgabeqdcqGHris5aOWaaWbaaSqabeaacqaIYaGmaaaaaa@42C1@/(*n *- 1), μ^=1k∑iy¯i·
 MathType@MTEF@5@5@+=feaafiart1ev1aaatCvAUfKttLearuWrP9MDH5MBPbIqV92AaeXatLxBI9gBaebbnrfifHhDYfgasaacH8akY=wiFfYdH8Gipec8Eeeu0xXdbba9frFj0=OqFfea0dXdd9vqai=hGuQ8kuc9pgc9s8qqaq=dirpe0xb9q8qiLsFr0=vr0=vr0dc8meaabaqaciaacaGaaeqabaqabeGadaaakeaaiiGacuWF8oqBgaqcaiabg2da9maalaaabaGaeGymaedabaGaem4AaSgaamaaqafabaGafmyEaKNbaebadaWgaaWcbaGaemyAaKMaeS4JPFgabeaaaeaacqWGPbqAaeqaniabggHiLdaaaa@3ADB@, and s2=1k∑isi2
 MathType@MTEF@5@5@+=feaafiart1ev1aaatCvAUfKttLearuWrP9MDH5MBPbIqV92AaeXatLxBI9gBaebbnrfifHhDYfgasaacH8akY=wiFfYdH8Gipec8Eeeu0xXdbba9frFj0=OqFfea0dXdd9vqai=hGuQ8kuc9pgc9s8qqaq=dirpe0xb9q8qiLsFr0=vr0=vr0dc8meaabaqaciaacaGaaeqabaqabeGadaaakeaacqWGZbWCdaahaaWcbeqaaiabikdaYaaakiabg2da9maalaaabaGaeGymaedabaGaem4AaSgaamaaqafabaGaem4Cam3aa0baaSqaaiabdMgaPbqaaiabikdaYaaaaeaacqWGPbqAaeqaniabggHiLdaaaa@3A05@. We therefore estimate the WSCV for binary assessments by:

ν^=s/y¯.
 MathType@MTEF@5@5@+=feaafiart1ev1aaatCvAUfKttLearuWrP9MDH5MBPbIqV92AaeXatLxBI9gBaebbnrfifHhDYfgasaacH8akY=wiFfYdH8Gipec8Eeeu0xXdbba9frFj0=OqFfea0dXdd9vqai=hGuQ8kuc9pgc9s8qqaq=dirpe0xb9q8qiLsFr0=vr0=vr0dc8meaabaqaciaacaGaaeqabaqabeGadaaakeaaiiGacuWF9oGBgaqcaiabg2da9iabdohaZjabc+caViqbdMha5zaaraGaeiOla4caaa@344D@

A case of special interest to clinical epidemiologists is when *n *= 2, or a test re-test reliability study involving two readings per subject. For this case we investigate the sample size issue in the following section.

## Results

### The special case n = 2

Under the above set-up, the common correlation model (CCM) (see Mak [15], Bloch and Kraemer [16]) provides an appropriate description for the joint distribution of (*Y*_*ij*_, *Y*_*il*_):

*P*_11 _= *P*(*Y*_*ij *_= 1,*Y*_*il *_= 1) = *π*^2 ^+ *ρπ*(1 - *π*).

*P*_10 _= *P*_01 _= *P*(*Y*_*ij *_= 1,*Y*_*il *_= 0) = *P*(*Y*_*ij *_= 0,*Y*_*il *_= 1) = (1 - *ρ*)*π*(1 - *π*).     (10)

*P*_00 _= *P*(*Y*_*ij *_= 0,*Y*_*il *_= 0) = (1 - *π*)^2 ^+ *ρπ*(1 - *π*).

The data layout can be summarized as in Table 8.

**Table 8:** Data layout for a 2 × 2 binary classification

		1^st ^measurement (*Y*_1_)	
		1	0
2^nd ^Measurement *Y*_2_	1	*k*_11_	*k*_01_
	0	*k*_10_	*k*_00_

where, *k *= *k*_11 _+ *k*_01 _+ *k*_10 _+ *k*_11_.

Since for the *i*^*th *^subject, the mean of two measurements is *μ*_*i *_= 12
 MathType@MTEF@5@5@+=feaafiart1ev1aaatCvAUfKttLearuWrP9MDH5MBPbIqV92AaeXatLxBI9gBaebbnrfifHhDYfgasaacH8akY=wiFfYdH8Gipec8Eeeu0xXdbba9frFj0=OqFfea0dXdd9vqai=hGuQ8kuc9pgc9s8qqaq=dirpe0xb9q8qiLsFr0=vr0=vr0dc8meaabaqaciaacaGaaeqabaqabeGadaaakeaadaWcaaqaaiabigdaXaqaaiabikdaYaaaaaa@2E9E@(*Y*_*i*1 _+ *Y*_*i*2_) when summed up over all subjects we have:

∑i=1kμi=12[(k11+k10)+(k11+k01)]
 MathType@MTEF@5@5@+=feaafiart1ev1aaatCvAUfKttLearuWrP9MDH5MBPbIqV92AaeXatLxBI9gBaebbnrfifHhDYfgasaacH8akY=wiFfYdH8Gipec8Eeeu0xXdbba9frFj0=OqFfea0dXdd9vqai=hGuQ8kuc9pgc9s8qqaq=dirpe0xb9q8qiLsFr0=vr0=vr0dc8meaabaqaciaacaGaaeqabaqabeGadaaakeaadaaeWbqaaGGaciab=X7aTnaaBaaaleaacqWGPbqAaeqaaaqaaiabdMgaPjabg2da9iabigdaXaqaaiabdUgaRbqdcqGHris5aOGaeyypa0ZaaSaaaeaacqaIXaqmaeaacqaIYaGmaaGaei4waSLaeiikaGIaem4AaS2aaSbaaSqaaiabigdaXiabigdaXaqabaGccqGHRaWkcqWGRbWAdaWgaaWcbaGaeGymaeJaeGimaadabeaakiabcMcaPiabgUcaRiabcIcaOiabdUgaRnaaBaaaleaacqaIXaqmcqaIXaqmaeqaaOGaey4kaSIaem4AaS2aaSbaaSqaaiabicdaWiabigdaXaqabaGccqGGPaqkcqGGDbqxaaa@5033@

Therefore, an unbiased estimator of the population mean *π *is:

μ^=12k[k10+k01+2k11],
 MathType@MTEF@5@5@+=feaafiart1ev1aaatCvAUfKttLearuWrP9MDH5MBPbIqV92AaeXatLxBI9gBaebbnrfifHhDYfgasaacH8akY=wiFfYdH8Gipec8Eeeu0xXdbba9frFj0=OqFfea0dXdd9vqai=hGuQ8kuc9pgc9s8qqaq=dirpe0xb9q8qiLsFr0=vr0=vr0dc8meaabaqaciaacaGaaeqabaqabeGadaaakeaaiiGacuWF8oqBgaqcaiabg2da9maalaaabaGaeGymaedabaGaeGOmaiJaem4AaSgaaiabcUfaBjabdUgaRnaaBaaaleaacqaIXaqmcqaIWaamaeqaaOGaey4kaSIaem4AaS2aaSbaaSqaaiabicdaWiabigdaXaqabaGccqGHRaWkcqaIYaGmcqWGRbWAdaWgaaWcbaGaeGymaeJaeGymaedabeaakiabc2faDjabcYcaSaaa@4341@

and the MSW, is *S*^2 ^= 12k
 MathType@MTEF@5@5@+=feaafiart1ev1aaatCvAUfKttLearuWrP9MDH5MBPbIqV92AaeXatLxBI9gBaebbnrfifHhDYfgasaacH8akY=wiFfYdH8Gipec8Eeeu0xXdbba9frFj0=OqFfea0dXdd9vqai=hGuQ8kuc9pgc9s8qqaq=dirpe0xb9q8qiLsFr0=vr0=vr0dc8meaabaqaciaacaGaaeqabaqabeGadaaakeaadaWcaaqaaiabigdaXaqaaiabikdaYiabdUgaRbaaaaa@2FFD@(*k*_10 _+ *k*_01_). Hence the sample coefficient of variation for binary responses is:

ν^=Sμ^=2k(k10+k01)k10+k01+2k11.     (11)
 MathType@MTEF@5@5@+=feaafiart1ev1aaatCvAUfKttLearuWrP9MDH5MBPbIqV92AaeXatLxBI9gBaebbnrfifHhDYfgasaacH8akY=wiFfYdH8Gipec8Eeeu0xXdbba9frFj0=OqFfea0dXdd9vqai=hGuQ8kuc9pgc9s8qqaq=dirpe0xb9q8qiLsFr0=vr0=vr0dc8meaabaqaciaacaGaaeqabaqabeGadaaakeaaiiGacuWF9oGBgaqcaiabg2da9maalaaabaGaem4uamfabaGaf8hVd0MbaKaaaaGaeyypa0ZaaSaaaeaadaGcaaqaaiabikdaYiabdUgaRjabcIcaOiabdUgaRnaaBaaaleaacqaIXaqmcqaIWaamaeqaaOGaey4kaSIaem4AaS2aaSbaaSqaaiabicdaWiabigdaXaqabaGccqGGPaqkaSqabaaakeaacqWGRbWAdaWgaaWcbaGaeGymaeJaeGimaadabeaakiabgUcaRiabdUgaRnaaBaaaleaacqaIWaamcqaIXaqmaeqaaOGaey4kaSIaeGOmaiJaem4AaS2aaSbaaSqaaiabigdaXiabigdaXaqabaaaaOGaeiOla4IaaCzcaiaaxMaadaqadaqaaiabigdaXiabigdaXaGaayjkaiaawMcaaaaa@5229@

Since *E*(μ^
 MathType@MTEF@5@5@+=feaafiart1ev1aaatCvAUfKttLearuWrP9MDH5MBPbIqV92AaeXatLxBI9gBaebbnrfifHhDYfgasaacH8akY=wiFfYdH8Gipec8Eeeu0xXdbba9frFj0=OqFfea0dXdd9vqai=hGuQ8kuc9pgc9s8qqaq=dirpe0xb9q8qiLsFr0=vr0=vr0dc8meaabaqaciaacaGaaeqabaqabeGadaaakeaaiiGacuWF8oqBgaqcaaaa@2E79@) = *π *and *E*(*SD*)= *π*(1 - *π*)(1 - *ρ*) we define ν=π−1(1−π)(1−ρ)
 MathType@MTEF@5@5@+=feaafiart1ev1aaatCvAUfKttLearuWrP9MDH5MBPbIqV92AaeXatLxBI9gBaebbnrfifHhDYfgasaacH8akY=wiFfYdH8Gipec8Eeeu0xXdbba9frFj0=OqFfea0dXdd9vqai=hGuQ8kuc9pgc9s8qqaq=dirpe0xb9q8qiLsFr0=vr0=vr0dc8meaabaqaciaacaGaaeqabaqabeGadaaakeaaiiGacqWF9oGBcqGH9aqpdaGcaaqaaiab=b8aWnaaCaaaleqabaGaeyOeI0IaeGymaedaaOGaeiikaGIaeGymaeJaeyOeI0Iae8hWdaNaeiykaKIaeiikaGIaeGymaeJaeyOeI0Iae8xWdiNaeiykaKcaleqaaaaa@3DE9@ as the population coefficient of variation for dichotomous outcome with its MLE given by ν^
 MathType@MTEF@5@5@+=feaafiart1ev1aaatCvAUfKttLearuWrP9MDH5MBPbIqV92AaeXatLxBI9gBaebbnrfifHhDYfgasaacH8akY=wiFfYdH8Gipec8Eeeu0xXdbba9frFj0=OqFfea0dXdd9vqai=hGuQ8kuc9pgc9s8qqaq=dirpe0xb9q8qiLsFr0=vr0=vr0dc8meaabaqaciaacaGaaeqabaqabeGadaaakeaaiiGacuWF9oGBgaqcaaaa@2E7B@. The CCM can be re-parameterized by substituting (1−ν21−ππ)
 MathType@MTEF@5@5@+=feaafiart1ev1aaatCvAUfKttLearuWrP9MDH5MBPbIqV92AaeXatLxBI9gBaebbnrfifHhDYfgasaacH8akY=wiFfYdH8Gipec8Eeeu0xXdbba9frFj0=OqFfea0dXdd9vqai=hGuQ8kuc9pgc9s8qqaq=dirpe0xb9q8qiLsFr0=vr0=vr0dc8meaabaqaciaacaGaaeqabaqabeGadaaakeaadaqadaqaaiabigdaXiabgkHiTmaalaaabaacciGae8xVd42aaWbaaSqabeaacqaIYaGmaaaakeaacqaIXaqmcqGHsislcqWFapaCaaGae8hWdahacaGLOaGaayzkaaaaaa@3857@ for the reliability coefficient *ρ*. Applying the delta method, the first order approximation to the variance of ν^
 MathType@MTEF@5@5@+=feaafiart1ev1aaatCvAUfKttLearuWrP9MDH5MBPbIqV92AaeXatLxBI9gBaebbnrfifHhDYfgasaacH8akY=wiFfYdH8Gipec8Eeeu0xXdbba9frFj0=OqFfea0dXdd9vqai=hGuQ8kuc9pgc9s8qqaq=dirpe0xb9q8qiLsFr0=vr0=vr0dc8meaabaqaciaacaGaaeqabaqabeGadaaakeaaiiGacuWF9oGBgaqcaaaa@2E7B@ is shown to be:

var(*∂*) = *k*^-1 ^(*a*_1 _+ *a*_2 _+ *a*_3_),

where *a*_1 _= *υ*^2^(1 - *υ*^2^*π*)(1 - *π *+ *υ*^2^*π*^2^)/*π*,

*a*_2 _= (1 - 2*πυ*^2^)^2^(1 - 2*π*^2^*υ*^2^)/8*π*^2^, and

*a*_3 _= *υ*^2^(1 - 2*υ*^2^*π*)(1 - *υ*^2^*π*).     (12)

We suggest an approximate (1 - *α*)100% confidence interval as ν^±zα/2var⁡(ν^)
 MathType@MTEF@5@5@+=feaafiart1ev1aaatCvAUfKttLearuWrP9MDH5MBPbIqV92AaeXatLxBI9gBaebbnrfifHhDYfgasaacH8akY=wiFfYdH8Gipec8Eeeu0xXdbba9frFj0=OqFfea0dXdd9vqai=hGuQ8kuc9pgc9s8qqaq=dirpe0xb9q8qiLsFr0=vr0=vr0dc8meaabaqaciaacaGaaeqabaqabeGadaaakeaaiiGacuWF9oGBgaqcaiabgglaXkabdQha6naaBaaaleaacqWFXoqycqGGVaWlcqaIYaGmaeqaaOWaaOaaaeaacyGG2bGDcqGGHbqycqGGYbGCcqGGOaakcuWF9oGBgaqcaiabcMcaPaWcbeaaaaa@3D4A@.

### Example 3

To illustrate the methodology discussed in this section, we use data from an investigation of mammography by Powell et al. [17] concerning the equivalence of film-screen (FS) and digital images (DI). Two readings were made on the presence/absence (1/0) of malignancy by each rater on the same set of *k *= 58 patients. The data and the results of the analysis are summarized in Table 9. Both methods seem to have the same levels of reliability in terms of ICC and WSCV. We note that the 95% confidence interval is somewhat relatively wide, and this may be due to the fact that the sample size is not large enough.

**Table 9:** Data analysis from a mammography study by Powell et al. (1999).

Rater	*k*_00_	*k*_10 _+ *k*_01_	*k*_11_	ρ^ MathType@MTEF@5@5@+=feaafiart1ev1aaatCvAUfKttLearuWrP9MDH5MBPbIqV92AaeXatLxBI9gBaebbnrfifHhDYfgasaacH8akY=wiFfYdH8Gipec8Eeeu0xXdbba9frFj0=OqFfea0dXdd9vqai=hGuQ8kuc9pgc9s8qqaq=dirpe0xb9q8qiLsFr0=vr0=vr0dc8meaabaqaciaacaGaaeqabaqabeGadaaakeaaiiGacuWFbpGCgaqcaaaa@2E83@	ν^ MathType@MTEF@5@5@+=feaafiart1ev1aaatCvAUfKttLearuWrP9MDH5MBPbIqV92AaeXatLxBI9gBaebbnrfifHhDYfgasaacH8akY=wiFfYdH8Gipec8Eeeu0xXdbba9frFj0=OqFfea0dXdd9vqai=hGuQ8kuc9pgc9s8qqaq=dirpe0xb9q8qiLsFr0=vr0=vr0dc8meaabaqaciaacaGaaeqabaqabeGadaaakeaaiiGacuWF9oGBgaqcaaaa@2E7B@	*SE*(ν^ MathType@MTEF@5@5@+=feaafiart1ev1aaatCvAUfKttLearuWrP9MDH5MBPbIqV92AaeXatLxBI9gBaebbnrfifHhDYfgasaacH8akY=wiFfYdH8Gipec8Eeeu0xXdbba9frFj0=OqFfea0dXdd9vqai=hGuQ8kuc9pgc9s8qqaq=dirpe0xb9q8qiLsFr0=vr0=vr0dc8meaabaqaciaacaGaaeqabaqabeGadaaakeaaiiGacuWF9oGBgaqcaaaa@2E7B@)	95% C.I.
DI	9	5	44	0.73	26%	0.061	(0.14,0.38)
FS	9	7	42	0.65	31%	0.064	(0.19,0.43)

Note that if the observed frequencies in the sample of *k *subjects are given as in Table 10, we can write a simpler estimator of the WSCV as ν^=2kn2
 MathType@MTEF@5@5@+=feaafiart1ev1aaatCvAUfKttLearuWrP9MDH5MBPbIqV92AaeXatLxBI9gBaebbnrfifHhDYfgasaacH8akY=wiFfYdH8Gipec8Eeeu0xXdbba9frFj0=OqFfea0dXdd9vqai=hGuQ8kuc9pgc9s8qqaq=dirpe0xb9q8qiLsFr0=vr0=vr0dc8meaabaqaciaacaGaaeqabaqabeGadaaakeaaiiGacuWF9oGBgaqcaiabg2da9maakaaabaGaeGOmaiJaem4AaSMaemOBa42aaSbaaSqaaiabikdaYaqabaaabeaaaaa@3465@/(*n*_2 _+ 2*n*_1_). To construct an estimate of the confidence interval on *υ*, the MLE of *ρ*, ρ^=1−n22kπ^(1−π^)
 MathType@MTEF@5@5@+=feaafiart1ev1aaatCvAUfKttLearuWrP9MDH5MBPbIqV92AaeXatLxBI9gBaebbnrfifHhDYfgasaacH8akY=wiFfYdH8Gipec8Eeeu0xXdbba9frFj0=OqFfea0dXdd9vqai=hGuQ8kuc9pgc9s8qqaq=dirpe0xb9q8qiLsFr0=vr0=vr0dc8meaabaqaciaacaGaaeqabaqabeGadaaakeaaiiGacuWFbpGCgaqcaiabg2da9iabigdaXiabgkHiTmaalaaabaGaemOBa42aaSbaaSqaaiabikdaYaqabaaakeaacqaIYaGmcqWGRbWAcuWFapaCgaqcaiabcIcaOiabigdaXiabgkHiTiqb=b8aWzaajaGaeiykaKcaaaaa@3D73@ and ν^
 MathType@MTEF@5@5@+=feaafiart1ev1aaatCvAUfKttLearuWrP9MDH5MBPbIqV92AaeXatLxBI9gBaebbnrfifHhDYfgasaacH8akY=wiFfYdH8Gipec8Eeeu0xXdbba9frFj0=OqFfea0dXdd9vqai=hGuQ8kuc9pgc9s8qqaq=dirpe0xb9q8qiLsFr0=vr0=vr0dc8meaabaqaciaacaGaaeqabaqabeGadaaakeaaiiGacuWF9oGBgaqcaaaa@2E7B@ should be substituted in equation (12) where, from Donner and Eliasziw [18]π^=n1+2n22k
 MathType@MTEF@5@5@+=feaafiart1ev1aaatCvAUfKttLearuWrP9MDH5MBPbIqV92AaeXatLxBI9gBaebbnrfifHhDYfgasaacH8akY=wiFfYdH8Gipec8Eeeu0xXdbba9frFj0=OqFfea0dXdd9vqai=hGuQ8kuc9pgc9s8qqaq=dirpe0xb9q8qiLsFr0=vr0=vr0dc8meaabaqaciaacaGaaeqabaqabeGadaaakeaaiiGacuWFapaCgaqcaiabg2da9maalaaabaGaemOBa42aaSbaaSqaaiabigdaXaqabaGccqGHRaWkcqaIYaGmcqWGUbGBdaWgaaWcbaGaeGOmaidabeaaaOqaaiabikdaYiabdUgaRbaaaaa@38D3@.

**Table 10:** Data Layout for the CCM

Category	Ratings	Frequency	Probability
1	(1,1)	*n*_1_	*P*_1_(*c*) = *π *(1 - *υ*^2^*π*)
2	(1,0) or (0,1)	*n*_2_	*P*_2_(*c*) = 2*π*^2^*υ*^2^
3	(0,0)	*n*_3_	*P*_3_(*c*) = 1 - *π *- *π*^2^*υ*^2^
Total		*k*	1

### Sample size estimation

#### Methods

There has been increasing attention given recently to estimation of sample size using a confidence interval rather than a significance testing approach (e.g. Gardner and Altman [19]). This is consistent with recent arguments made by many authors, including Goodman and Berlin [20] who state that "confidence intervals should play an important role when setting sample size" and that " the size of a confidence interval can be predicted in the planning stages of an experiment and this can be a great help in understanding the implications of different sample size choices".

For comparative interest we also present sample size requirements needed to test *H*_0 _:*υ *= *υ*_0_, where *υ*_0 _is some hypothesized value of the WSCV *υ*.

### Fixed width confidence interval (CI) on *υ*

Following the approach described in Section 3.2, the approximate width of an (1 - *α*)100% CI on *υ *is, 2*z*_*α*/2_{var(ν^
 MathType@MTEF@5@5@+=feaafiart1ev1aaatCvAUfKttLearuWrP9MDH5MBPbIqV92AaeXatLxBI9gBaebbnrfifHhDYfgasaacH8akY=wiFfYdH8Gipec8Eeeu0xXdbba9frFj0=OqFfea0dXdd9vqai=hGuQ8kuc9pgc9s8qqaq=dirpe0xb9q8qiLsFr0=vr0=vr0dc8meaabaqaciaacaGaaeqabaqabeGadaaakeaaiiGacuWF9oGBgaqcaaaa@2E7B@)}^1/2^. An approximate sample size that yields an (1 - *α*)100% CI for *υ *with a desired width *w *obtains by setting *w *= 2*z*_*α*/2_{var(ν^
 MathType@MTEF@5@5@+=feaafiart1ev1aaatCvAUfKttLearuWrP9MDH5MBPbIqV92AaeXatLxBI9gBaebbnrfifHhDYfgasaacH8akY=wiFfYdH8Gipec8Eeeu0xXdbba9frFj0=OqFfea0dXdd9vqai=hGuQ8kuc9pgc9s8qqaq=dirpe0xb9q8qiLsFr0=vr0=vr0dc8meaabaqaciaacaGaaeqabaqabeGadaaakeaaiiGacuWF9oGBgaqcaaaa@2E7B@)}^1/2 ^and then solving for *k*:

*k *= 4zα/22
 MathType@MTEF@5@5@+=feaafiart1ev1aaatCvAUfKttLearuWrP9MDH5MBPbIqV92AaeXatLxBI9gBaebbnrfifHhDYfgasaacH8akY=wiFfYdH8Gipec8Eeeu0xXdbba9frFj0=OqFfea0dXdd9vqai=hGuQ8kuc9pgc9s8qqaq=dirpe0xb9q8qiLsFr0=vr0=vr0dc8meaabaqaciaacaGaaeqabaqabeGadaaakeaacqWG6bGEdaqhaaWcbaacciGae8xSdeMaei4la8IaeGOmaidabaGaeGOmaidaaaaa@32C6@(*a*_1 _+ *a*_2 _+ *a*_3 _)/*w*^2^.     (13)

### Hypothesis testing procedure

Donner and Eliasziw (DE) [18] developed the Goodness-of-fit (GOF) to efficiently construct a confidence interval and to estimate the sample size required to test a specific hypothesis on intra-class kappa value. Here we use the GOF to estimate the sample size needed for ensuring enrollment of a sufficient number of subjects in a reproducibility study. This follows from the observation that, to test the null hypothesis, *H*_0 _: *υ *= *υ*_0 _then:

χG2=∑l=13[nl−kP^l(ν0)]2kP^l(ν0)     (14)
 MathType@MTEF@5@5@+=feaafiart1ev1aaatCvAUfKttLearuWrP9MDH5MBPbIqV92AaeXatLxBI9gBamXvP5wqSXMqHnxAJn0BKvguHDwzZbqegyvzYrwyUfgarqqtubsr4rNCHbGeaGqiA8vkIkVAFgIELiFeLkFeLk=iY=Hhbbf9v8qqaqFr0xc9pk0xbba9q8WqFfeaY=biLkVcLq=JHqVepeea0=as0db9vqpepesP0xe9Fve9Fve9GapdbaqaaeGacaGaaiaabeqaamqadiabaaGcbaacciGae83Xdm2aa0baaSqaaiabdEeahbqaaiabikdaYaaakiabg2da9maaqahabaWaaSaaaeaacqGGBbWwcqWGUbGBdaWgaaWcbaGaemiBaWgabeaakiabgkHiTiabdUgaRjqbdcfaqzaajaWaaSbaaSqaaiabdYgaSbqabaGccqGGOaakcqWF9oGBdaWgaaWcbaGaeGimaadabeaakiabcMcaPiabc2faDnaaCaaaleqabaGaeGOmaidaaaGcbaGaem4AaSMafmiuaaLbaKaadaWgaaWcbaGaemiBaWgabeaakiabcIcaOiab=17aUnaaBaaaleaacqaIWaamaeqaaOGaeiykaKcaaaWcbaGaemiBaWMaeyypa0JaeGymaedabaGaeG4mamdaniabggHiLdGccaWLjaGaaCzcamaabmaabaGaeGymaeJaeGinaqdacaGLOaGaayzkaaaaaa@664B@

has a non-central chi-square distribution with one degree of freedom under the alternative hypothesis *H*_1 _: *υ *= *υ*_1 _with non-centrality parameter

η=k∑l=13[Pl(ν1)−Pl(ν0)]2Pl(ν0).     (15)
 MathType@MTEF@5@5@+=feaafiart1ev1aaatCvAUfKttLearuWrP9MDH5MBPbIqV92AaeXatLxBI9gBaebbnrfifHhDYfgasaacH8akY=wiFfYdH8Gipec8Eeeu0xXdbba9frFj0=OqFfea0dXdd9vqai=hGuQ8kuc9pgc9s8qqaq=dirpe0xb9q8qiLsFr0=vr0=vr0dc8meaabaqaciaacaGaaeqabaqabeGadaaakeaaiiGacqWF3oaAcqGH9aqpcqWGRbWAdaaeWbqaamaalaaabaGaei4waSLaemiuaa1aaSbaaSqaaiabdYgaSbqabaGccqGGOaakcqWF9oGBdaWgaaWcbaGaeGymaedabeaakiabcMcaPiabgkHiTiabdcfaqnaaBaaaleaacqWGSbaBaeqaaOGaeiikaGIae8xVd42aaSbaaSqaaiabicdaWaqabaGccqGGPaqkcqGGDbqxdaahaaWcbeqaaiabikdaYaaaaOqaaiabdcfaqnaaBaaaleaacqWGSbaBaeqaaOGaeiikaGIae8xVd42aaSbaaSqaaiabicdaWaqabaGccqGGPaqkaaaaleaacqWGSbaBcqGH9aqpcqaIXaqmaeaacqaIZaWma0GaeyyeIuoakiabc6caUiaaxMaacaWLjaWaaeWaaeaacqaIXaqmcqaI1aqnaiaawIcacaGLPaaaaaa@5777@

Following DE it can be shown that the sample size needed to conduct a two-sided test with significance level *α *and power 1 - *β *is:

k=(z1−α/2+z1−β)2π2(ν12−ν02)2[(1−ν02π)−1+2ν0−2+π2(1−π−π2ν02)−1]−1     (16)
 MathType@MTEF@5@5@+=feaafiart1ev1aaatCvAUfKttLearuWrP9MDH5MBPbIqV92AaeXatLxBI9gBaebbnrfifHhDYfgasaacH8akY=wiFfYdH8Gipec8Eeeu0xXdbba9frFj0=OqFfea0dXdd9vqai=hGuQ8kuc9pgc9s8qqaq=dirpe0xb9q8qiLsFr0=vr0=vr0dc8meaabaqaciaacaGaaeqabaqabeGadaaakeaacqWGRbWAcqGH9aqpdaWcaaqaaiabcIcaOiabdQha6naaBaaaleaacqaIXaqmcqGHsisliiGacqWFXoqycqGGVaWlcqaIYaGmaeqaaOGaey4kaSIaemOEaO3aaSbaaSqaaiabigdaXiabgkHiTiab=j7aIbqabaGccqGGPaqkdaahaaWcbeqaaiabikdaYaaaaOqaaiab=b8aWnaaCaaaleqabaGaeGOmaidaaOGaeiikaGIae8xVd42aa0baaSqaaiabigdaXaqaaiabikdaYaaakiabgkHiTiab=17aUnaaDaaaleaacqaIWaamaeaacqaIYaGmaaGccqGGPaqkdaahaaWcbeqaaiabikdaYaaaaaGccqGGBbWwcqGGOaakcqaIXaqmcqGHsislcqWF9oGBdaqhaaWcbaGaeGimaadabaGaeGOmaidaaOGae8hWdaNaeiykaKYaaWbaaSqabeaacqGHsislcqaIXaqmaaGccqGHRaWkcqaIYaGmcqWF9oGBdaqhaaWcbaGaeGimaadabaGaeyOeI0IaeGOmaidaaOGaey4kaSIae8hWda3aaWbaaSqabeaacqaIYaGmaaGccqGGOaakcqaIXaqmcqGHsislcqWFapaCcqGHsislcqWFapaCdaahaaWcbeqaaiabikdaYaaakiab=17aUnaaDaaaleaacqaIWaamaeaacqaIYaGmaaGccqGGPaqkdaahaaWcbeqaaiabgkHiTiabigdaXaaakiabc2faDnaaCaaaleqabaGaeyOeI0IaeGymaedaaOGaaCzcaiaaxMaadaqadaqaaiabigdaXiabiAda2aGaayjkaiaawMcaaaaa@7B08@

where z_1-*α*/2 _and z_1-*β *_are the critical values of the standard normal distribution corresponding to *α *and *β*.

As an example suppose it is of interest to test *H*_0_: *v *= 0.04 versus *H*_1_: *v *= 0.1, where *v*_0 _corresponds to high reliability. To ensure with 80 per sent probability a significant result at *α *= 5% and *π *= 0.30 when *v*_1 _= 0.10, we compute the required number of subjects from the above equation as *k *= 986 and when *π = *0.50, *k *= 355. For the sake of comparison to the fixed width CI procedure, suppose it is of interest to construct 95% CI on *v *with expected width *w *= 0.10. If the hypothesized values of *v *is 0.10 and *π = *0.30, then from (13) *k *= 1100, and if *π = *0.5, then *k *= 400.

## Discussion

The ICC has been traditionally used to assess the reliability of a measurement. QS considered the WSCV as an alternative measure of reproducibility for continuous scale measurements. It should be emphasized that our investigation has not allowed for forms of systematic error (e.g. measurement, or trend that is unaccounted for in the model). A reviewer of this paper indicated that this is beyond our scope. In this paper we have dealt with the issue of sample size estimation of the WSCV from continuous and binary scale measurements focusing on random measurement error, in the conventional way that reliability is usually discussed.

As in any reliability study, a crucial decision that a researcher faces in the design stage is the determination of the number of subjects, k and the number of measurements per subject, n. We have discussed two alternative statistical techniques to determine an optimal allocation. When we have prior knowledge of what constitutes an acceptable level of reproducibility, a hypothesis testing approach may be used. We used this approach in the case of binary outcome variable, following the GOF approach proposed by DE. The application of the GOF was straightforward because the number of replicates n = 2 was fixed. However, there are situations, when appropriate values of the reliability coefficient under the null and alternative hypotheses may be difficult to specify. An alternative to hypotheses testing is the efficient allocation of the sample, and the guidelines provided in this article for the continuous scale measurements allow selection of the pair (*n*, *k*) that maximizes the precision of the estimated coefficient under cost constrains. We note that cost implications, for dichotomous assessments, are quite important particularly when *n *is larger than two, which we intend to report on in a future paper.

Finally it is noted that in practice, the optimal allocation must be integer values, and the net loss/gain in precision as a result of rounding the values the values of (n, k) was negligible. Ideally one should adopt one of the available optimization algorithms, often referred to as integer programming models. These models are suited for the optimal allocations problems since the main concern was to find the best solution(s) in a well-defined discrete space.

## Conclusion

The WSCV is a useful index measure of measurements reliability. Investigators may design reliability studies using either efficiency or cost considerations. For continuous measurements, optimal allocation of the sample may be achieved with as few as two replications per subject. For dichotomous data, when each subject is measured twice, investigators may use, either fixed length confidence interval, or power considerations is estimating the sample size. Both methods produce comparable results.

## Competing interests

The author(s) declare that they have no competing interests.

The authors contributed equally to this work

## Pre-publication history

The pre-publication history for this paper can be accessed here:


